# Stiffness-dependent alveolar type II cell senescence in idiopathic pulmonary fibrosis

**DOI:** 10.1186/s12964-026-02881-5

**Published:** 2026-04-25

**Authors:** Chih-Ru Lin, Khanutsanan Woranam, Hassan Hayek, Jonathan Jeger, Loukmane Karim, Beata Kosmider, Rafal Kaminski, Christopher W. Schultz, Sudhir Bolla, Nathaniel Marchetti, Gerard J. Criner, Karim Bahmed

**Affiliations:** 1 Department of Biochemistry, School of Medicine, College of Medicine, Kaohsiung Medical University, Kaohsiung, Taiwan; 2 Center for Inflammation and Lung Research, Lewis Katz School of Medicine, Temple University, Philadelphia, PA 19140 USA; 3 Department of Microbiology, Immunology, and Inflammation, Lewis Katz School of Medicine, Temple University, Philadelphia, PA 19140 USA; 4 Center for Neurovirology and Gene Editing, Lewis Katz School of Medicine, Temple University, Philadelphia, PA 19140 USA; 5 Department of Cancer and Cellular Biology, Lewis Katz School of Medicine, Temple University, Philadelphia, PA 19140 USA; 6 Department of Thoracic Medicine and Surgery, Lewis Katz School of Medicine, Temple University, Philadelphia, PA 19140 USA

**Keywords:** IPF, ATII cells, DNA damage, DNA repair, Mechanical stress

## Abstract

**Background:**

IPF is characterized by the fibrotic response involving abnormally activated ATII cells. A stiffened matrix exerts extrinsic microenvironment-derived forces, leading to mechanical stress and ATII cell dysfunction. However, these mechanisms remain largely unknown.

**Methods:**

ATII cells were isolated from control organ donors and the explanted lungs of patients with IPF. DNA damage was assessed by the comet assay and western blotting. ATII cell senescence was determined by β-galactosidase staining. TERRA transcripts, the long noncoding RNA telomeric repeat-containing RNA, and R-loops, which can impact genome integrity, were analyzed by qPCR. H3K27me3 and H3K9me3 levels were assessed by immunofluorescence. ATII cells were cultured on PDMS hydrogel to study mechanical stiffness. The impact of YAP/TAZ inhibition on control ATII cells cultured on a 50 kPa PDMS hydrogel was also evaluated.

**Results:**

A significant increase in γH2AX expression and impairment of NHEJ were detected in ATII cells in IPF. The data obtained indicate defective DDR and decreased DNA damage repair capacity in ATII cells in patients with this disease, which can contribute to senescence, replication stress, transcription replication conflicts, and genomic instability. Moreover, the increased levels of H3K27me3 and H3K9me3 in ATII cells in IPF suggests repressed transcriptional activity. Nuclear lamina discontinuity was identified by immunofluorescence using emerin staining. Mechanotransduction signaling influenced epigenetic regulation in ATII cells in response to mechanical stiffness.

**Conclusion:**

This study provides new mechanistic insights into the function of ATII cells and the integrity of the alveolar epithelium under highly stressed conditions in IPF. The data underscores the importance of mechanical stiffness on ATII cell dysfunction and senescence.

**Supplementary Information:**

The online version contains supplementary material available at 10.1186/s12964-026-02881-5.

## Background

Lung cells are constantly exposed to environmental and endogenous factors. Alveolar epithelium is composed of alveolar type I (ATI) and alveolar type II (ATII) cells [1]. ATI cells cover 95–98% of the alveolar epithelium, are large, squamous, and function as the epithelial component of the air-blood barrier [2–5]. In contrast, ATII cells represent 2–5% of the alveolar epithelium. They have a stem cell potential, proliferate, and differentiate into ATI cells [6, 7].

Idiopathic pulmonary fibrosis (IPF) is a fatal disease with a median survival time of 2–5 years from diagnosis [8]. The mechanisms underlying its development and progression remain poorly understood. The IPF microenvironment is partially characterized by the accumulation of extracellular matrix (ECM) proteins in the interstitial space and increased stiffness [9]. This stiffened matrix exerts extrinsic microenvironment-derived forces on ATII cells, leading to mechanical stress [10]. We have reported impaired ATII cell function in IPF [11, 12]. Cells can respond to external forces by modulating cytoskeletal organization [13]. The nuclear envelope separates the nucleus from the cytoplasm, and lamins provide structural support, connecting the nucleus to the cytoskeleton [14]. Cytosolic mechanical tensions affect the physical properties of the nucleus. This can lead to the activation and nuclear translocation of mechanical sensor proteins, such as transcription factor yes-associated protein (YAP) [15].

Damaged DNA generates replication errors, mutations, and genomic instability [16, 17]. It can cause persistent anomalies in post-mitotic and irreplaceable cells unless repaired by efficient mechanisms. DNA double-strand breaks (DSBs) are the most harmful and dangerous type of DNA lesion [18]. Failure to accurately repair DSBs can result in the loss or gain of genetic information through several mechanisms, including deletions, insertions, or chromosomal translocations [19]. This can lead to damage-induced cellular alterations and contribute to disease development by impairing DNA repair, causing genomic instability, and promoting the accumulation of genetic aberrations [20, 21]. A better understanding of the mechanisms regulating DNA damage and repair could lead to the identification of novel treatment targets to prevent and combat diseases. Non-homologous end-joining (NHEJ), an error-prone mechanism, and homologous recombination (HR), a high-fidelity DNA repair mechanism, are the primary repair pathways for resolving DSBs [22]. NHEJ is predominantly utilized in vertebrates [23]. It occurs throughout the cell cycle, whereas HR repair is restricted to the S/G2 phases, since complementary homologous sequences are required [24]. An early event in classical NHEJ is initiated when a Ku70/80 heterodimer binds to DSBs, which recruits DNA-dependent protein kinase catalytic subunit (DNA-PKcs) and other DNA repair proteins [25]. Various proteins have also been shown to play roles in NHEJ, including TDP1, RTT109, EXO1, PPH3, and POL3 [26–30].

DNA damage repair (DDR) machinery resolves a variety of DNA lesions in human cells. Aberrant DDR signaling leads to genomic instability [31]. Upon DNA damage, Ataxia-Telangiectasia Mutated (ATM) and Ataxia-Telangiectasia and Rad3-related (ATR) stimulate the DDR cascade, followed by the recruitment of various downstream DNA repair proteins [32–35]. H2AX is phosphorylated at serine 139 (γH2AX), forming a dock to recruit DDR-related proteins. Activation of P53 leads to transient cell cycle arrest, providing time for DNA repair. The recruitment of distinct proteins to DSB sites is a critical step that promotes a specific repair pathway. However, unrepaired DNA damage induces permanent cell cycle arrest, senescence, or apoptosis and is associated with numerous diseases.

The relationship between mechanical stress, DNA damage repair, and ATII cell senescence is still understudied. Additionally, nuclear mechanical stress in ATII cells in IPF remains poorly characterized. Here, ATII cells were isolated from patients with this disease and control organ donors. We identified defective DDR, leading to incomplete DNA damage repair and cell senescence.

## Methods

### Human subjects

Control lungs were obtained from 44 de-identified organ donors (20 males and 24 females, age 64 ± 10 years) without a history of chronic lung disease, a clinical history of infection, or prolonged ventilator support. They were obtained through the Gift of Life Donor Program (Philadelphia, PA). Lungs from 38 IPF patients (31 males and 7 females, age 63 ± 8 years) were from the Temple Biobank (Temple University, Philadelphia, PA). ATII cells were isolated, with high purity, as we previously described [36]. Briefly, the lung was minced and a cell suspension was obtained. The cells were filtered and purified using a density gradient made of Optiprep (Sigma-Aldrich, St. Louis, MO) and EpCAM microbeads (Miltenyi Biotec, Germany) for a positive selection.

### Immunofluorescence

The following antibodies were used for immunofluorescence: SP-C, H3K9me3, dynein, dsDNA, P16, P21, YAP, and topoisomerase II (Santa Cruz Biotechnology), HTII-280 (Terrace Biotech), SP-A, XLF, and DNA ligase IV (Novus), lamin B and SP-C (Proteintech), S9.6 (Absolute Antibody), Ku70/80 (Abcam), emerin, H3K27me3, α-SMA, and γH2AX (Abclonal). Control IgG staining was performed using mouse, rabbit, and goat IgG (Bioss) (Fig. S1). Emerin discontinuity at the lamina was assessed [37]. The corresponding secondary antibodies, Alexa Fluor 488, Alexa Fluor 594, or Alexa Fluor 647 (Invitrogen), were applied for 1 h. Fluoroshield mounting medium with DAPI (Abcam) was used to stain nuclei. Images were captured using a confocal laser-scanning microscope (Zeiss). For mechanical stiffness experiments, images were taken using fluorescence microscopy (Olympus IX71). Pearson's correlation coefficient was used to analyze protein co-localization. Fluorescence intensity was analyzed and quantified using ImageJ software (NIH).

### DCF staining

2’−7’-Dichlorodihydrofluorescein diacetate (DCF-DA) is converted to a highly fluorescent DCF compound by reaction with reactive oxygen species (ROS). Freshly isolated ATII cells were used for incubation with the DCF-DA probe (Sigma-Aldrich), as we previously described, with slight modifications [38]. Briefly, 5 × 10^5^ ATII cells were incubated with 5 μM DCF-DA for 20 min at 37 ^o^C, then washed with PBS. DCF fluorescence intensity was analyzed using the LSR-II flow cytometer (BD Biosciences) and FlowJo software (Tree Star, Inc).

### Comet assay

DNA damage and recovery were analyzed in ATII cells isolated from control organ donors and IPF patients using the OxiSelect Comet Assay (Cell Biolabs) according to the manufacturer's instructions. ATII cells were also treated with 50 μM etoposide for 24 h, followed by media removal to determine repair capacity after 24 h [39]. Briefly, ATII cells were immobilized in low-melting-point agarose on CometSlides, then lysed. The slides were subjected to alkaline electrophoresis for 30 min, washed, dried, and stained with a Vista Green DNA Dye. Images were taken using fluorescence microscopy (Zeiss Axioskop 2) and analyzed by OpenComet software [40]. Comet assay images were analyzed to quantify DNA damage using the Olive tail moment (OTM) parameter. Fluorescent images of individual comets were captured under identical exposure settings. For each nucleus, the software automatically defined the comet head and tail regions based on fluorescence intensity thresholds. The OTM was calculated as the percentage of total DNA in the tail and the distance between the centers of mass of head and tail DNA distributions: OTM = (% tail DNA x Tail moment length), representing the intensity-weighted centers of gravity of DNA. This measurement reflects both the extent of DNA migration and the amount of fragmented DNA, providing a sensitive indicator of DNA strand breaks. For each sample, 75–100 randomly selected, non-overlapping comets were analyzed.

### PDMS hydrogel substrates

Mechanical stiffness was simulated using Dow Corning Sylgrad 527 silicone dielectric gel (Ellsworth) [41]. Briefly, the components of the polydimethylsiloxane (PDMS) hydrogel were mixed at different ratios to achieve the desired tensions. The ratio of components was 1.2 for the 2 kPa hydrogel mimicking soft tissue, and 0.3 for the 50 kPa hydrogel reflecting fibrotic tissue. Plates were coated with 67 μl hydrogel and incubated overnight at 60 °C, then coated with a thin layer of a mixture of 20% Matrigel (BD Biosciences) and 80% rat-tail collagen (Corning). Freshly isolated ATII cells were cultured in DMEM supplemented with 10% FBS, 2 mM glutamine, 10 μg/ml gentamicin, 100U/ml penicillin, 100 μg/ml streptomycin, and 2.5 μg/ml amphotericin B (Thermo Scientific) for 24 h or 48 h [42]. Cells were treated with 2.5 nM YAP/TAZ inhibitor-2 (MedChemExpress) for 48 h.

### Western blotting

Lung tissue and cell lysates were used to determine protein expression by western blotting, as we described previously, with slight modifications [38, 43]. The lysates were separated on 8–16% gradient gels (ThermoFisher Scientific) and transferred to nitrocellulose membranes. Protein concentrations were quantified using a BCA protein assay (ThermoFisher Scientific). The following antibodies were applied: 4-HNE (R&D Systems), P16, P21, P53, DJ-1, SRX, TDP-1, RAD50, RAD51, NBS1, XRCC4, DNA ligase III, dynein (Santa Cruz Biotechnology), MRE11 (Proteintech), SIRT6, PARP1 (Cell Signaling), γH2AX (Millipore), Ku80, XLF, DNA ligase IV (Novus), tubulin, β-actin (Sigma), and GAPDH (Abcam). The corresponding horseradish peroxidase (HRP)-conjugated AffiniPure secondary antibodies were purchased from Jackson ImmunoResearch. The blots were developed using an enhanced chemiluminescence western blotting kit (Millipore) according to the manufacturer's instructions. Images were quantified using ImageJ software (NIH).

### qPCR

Total RNA was isolated from ATII cells obtained from control organ donors and IPF patients using Quick-RNA MiniPrep (Zymo Research) according to the manufacturer's instructions. qPCR experiments were performed according to established good practices for mRNA analysis. RNA purity and integrity were confirmed prior to cDNA synthesis. RNA was reverse-transcribed into cDNA using the SuperScript IV First-Strand Synthesis System (ThermoFisher Scientific). Gene levels were determined by RT-PCR using SYBR Green Master Mix (ThermoFisher Scientific) and StepOnePlus Real-Time PCR System (Applied Biosystems). Primer sequences are shown in Table S1. Data were normalized to GAPDH and analyzed by the ΔCt method. Telomere length was measured using qPCR [44].

### Cell senescence

A β-galactosidase staining kit (Cell Signaling Technology) was applied to identify senescent cells. Freshly isolated ATII obtained from control organ donors and IPF patients were used and stained per the manufacturer’s instructions. Briefly, β-galactosidase staining solution was used at 37 °C overnight. The cells were imaged using the brightfield microscope (Olympus).

### Cell transfection

To study the restoration of ATII cell function in IPF, RNase H1 transfection was performed. RNase H1-GFP or GFP vectors were applied [45]. Freshly isolated ATII cells obtained from IPF were transfected by electroporation using the 4D-Nucleofector X Unit (Lonza). The cells were then cultured for 48 h as described above.

### Statistical analysis

Data are expressed as the means ± SD. Statistically significant differences among experimental groups were determined using a *t*-test or a one-way ANOVA. The value of *p* < 0.05 was considered significant. The results obtained were normalized to 1 and compared to the controls.

## Results

### ATII cell senescence in IPF

**Fig. 1:**
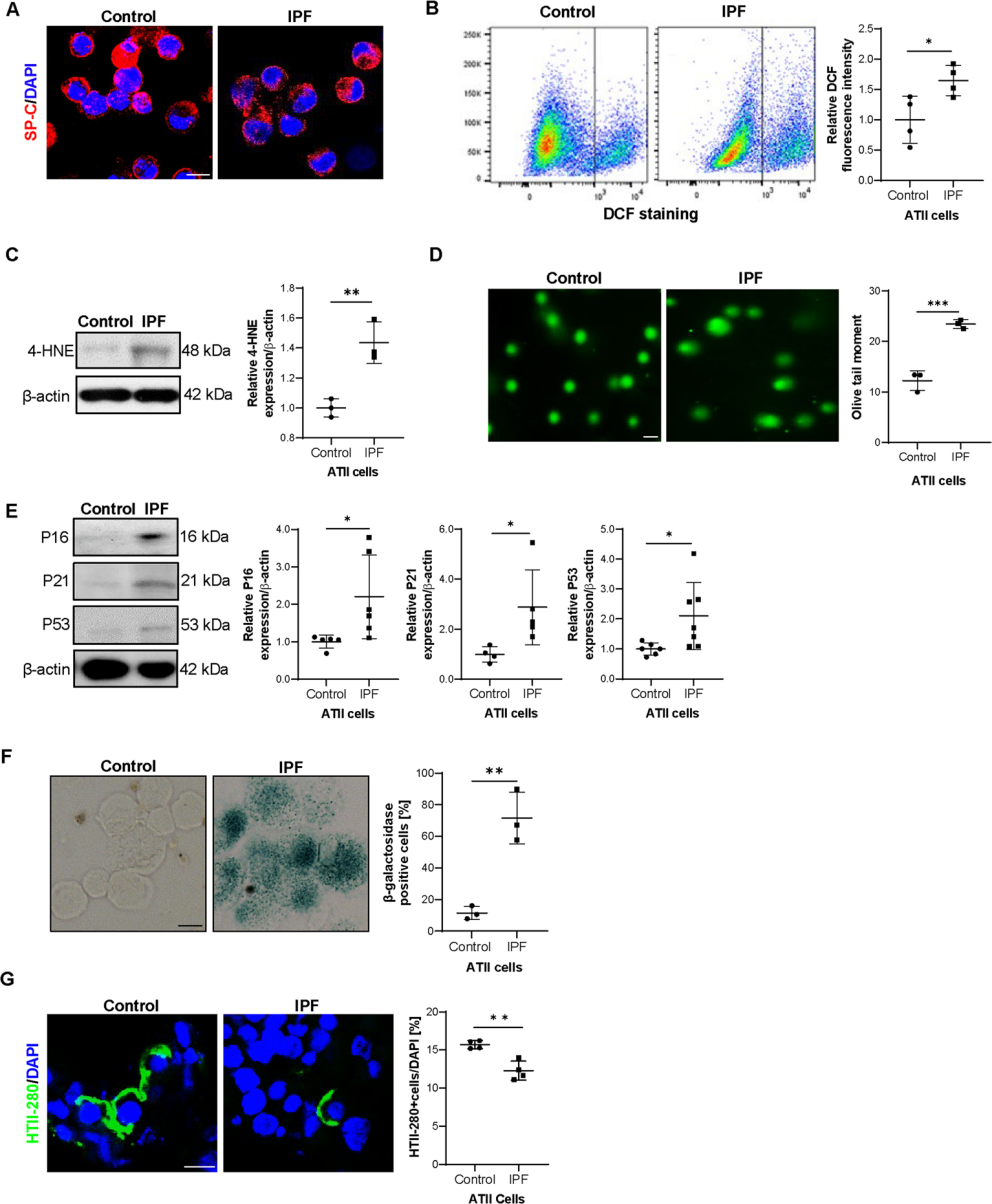
High oxidative stress and DNA damage in ATII cells in IPF patients. Lungs were obtained from control organ donors and IPF patients. **A** The purity of freshly isolated ATII cells using cell cytospins with SP-C staining by immunofluorescence. **B** ROS generation in ATII cells was analyzed using DCF by flow cytometry. The quantification of DCF fluorescence intensity is shown. **C** 4-HNE expression was determined by western blotting and normalized to β-actin. The quantification is shown. **D** DNA damage was evaluated by a comet assay. Olive tail moments are also shown. **E** P16, P21, and P53 levels were determined by western blotting and normalized to β-actin. The protein levels were quantified. **F** Freshly isolated ATII cells from control and IPF were used to analyze β-galactosidase activity. The quantification is shown. **G** Human lung tissue sections were stained with the HTII-280 antibody using immunofluorescence. The percentage of HTII-280 positive cells is shown. Data are normalized to control and expressed as means ± SD (scale bar—5 µm). *N* = 3–7 lungs per group; *p < 0.05; **p < 0.01; ***p < 0.001

We previously showed ATII cell dysfunction in IPF with a reduced ability to differentiate into ATI cells [12]. To understand the underlying mechanisms, freshly isolated ATII cells from control organ donors and IPF patients were used. We performed immunofluorescence staining with an SP-C antibody (Fig. 1A). Various mechanisms protect cells from ROS-induced damage. DCF fluorescence intensity was measured by flow cytometry to assess ROS levels in ATII cells from IPF and control organ donors. We found that ATII cells isolated from patients with this disease had higher levels than control organ donors (Fig. 1B). The expression of 4-HNE, a marker of lipid peroxidation products and oxidative stress, was analyzed by western blotting. Its levels were significantly higher in ATII cells isolated from IPF compared to controls (Fig. 1C). Since higher ROS and oxidative stress levels were detected in IPF, we analyzed DNA damage in freshly isolated ATII cells by comet assay. Compared to control cells, ATII cells obtained from IPF had higher Olive tail moments (Fig. 1D). These observations suggest oxidative stress-induced DNA damage and inefficient repair.

A compromised DNA repair system or irreparable DNA damage, associated with prolonged DDR signaling, can lead to permanent cell proliferative arrest and senescence [46]. ATII cells obtained from IPF exhibited marked cellular senescence, as indicated by elevated expressions of P21 and P16. The levels of P53 were also analyzed, and we found that its expression increased in ATII cells obtained from IPF compared to controls (Fig. 1E). In addition, we found that the percentage of β-galactosidase-positive ATII cells increased in patients with this disease (Fig. 1F). Moreover, the percentage of ATII cells in lung tissue obtained from IPF patients was significantly decreased compared to controls (Fig. 1G). Together, these data suggest that sustained unrepaired DNA damage leads to elevated levels of P16, P53, and P21 and to DNA damage–initiated cellular senescence. These findings support the critical role of impaired ATII cell DNA damage repair in the development of IPF.

### Inefficient antioxidant defense system in IPF

**Fig. 2:**
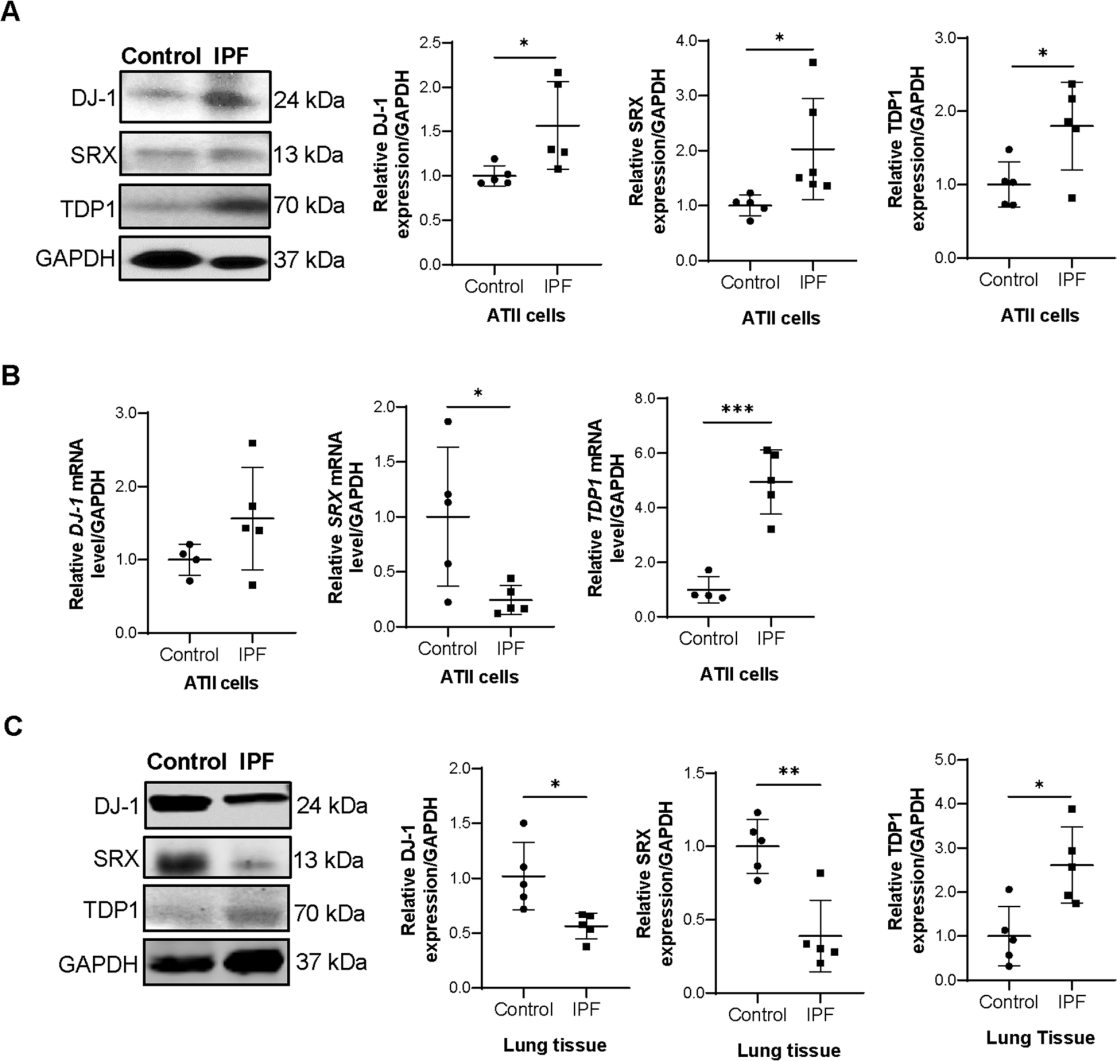
Antioxidant response in ATII cells in IPF. Protein and gene expression were analyzed in lung tissue and in ATII cells obtained from control organ donors and IPF patients. **A** The protein levels of DJ-1, SRX, and TDP1 were determined in ATII cells by western blotting. The quantification is also shown. **B** *DJ-1*, *SRX,* and *TDP1* mRNA expression was analyzed in ATII cells by RT-PCR. **C** DJ-1, SRX, and TDP-1 protein levels in lung tissue were analyzed by western blotting. Data are normalized to controls and expressed as means ± SD. *N* = 4–6 lungs per group.*p < 0.05; **p < 0.01; ***p < 0.001

An impairment of the antioxidant system's efficiency can increase cell susceptibility to oxidative stress and contribute to DNA damage [47]. DJ-1, SRX and TDP1 protect from oxidative stress-induced DNA damage [48–50]. Their expression was upregulated in freshly isolated ATII cells from IPF compared to controls (Fig. 2A). The mRNA expression of these genes was also determined. *TDP1* expression was increased, and *SRX* levels decreased in IPF (Fig. 2B). No significant differences in *DJ-1* levels were detected in the analyzed groups. We also used lung tissue from IPF and control organ donors. DJ-1 and SRX expression were decreased, and TDP1 levels were increased in this disease (Fig. 2C). It is worth noting that although DJ-1, SRX, and TDP1 protein levels were elevated in ATII cells in IPF, oxidative stress and DNA damage were also increased. This suggests that the activation of genes conferring cellular cytoprotection was inefficient in preventing cell damage. The discrepancy between results obtained from ATII cells and lung tissue may be due to responses from multiple cell types in the latter sample.

### DNA damage repair pathways in ATII cells in IPF

**Fig. 3:**
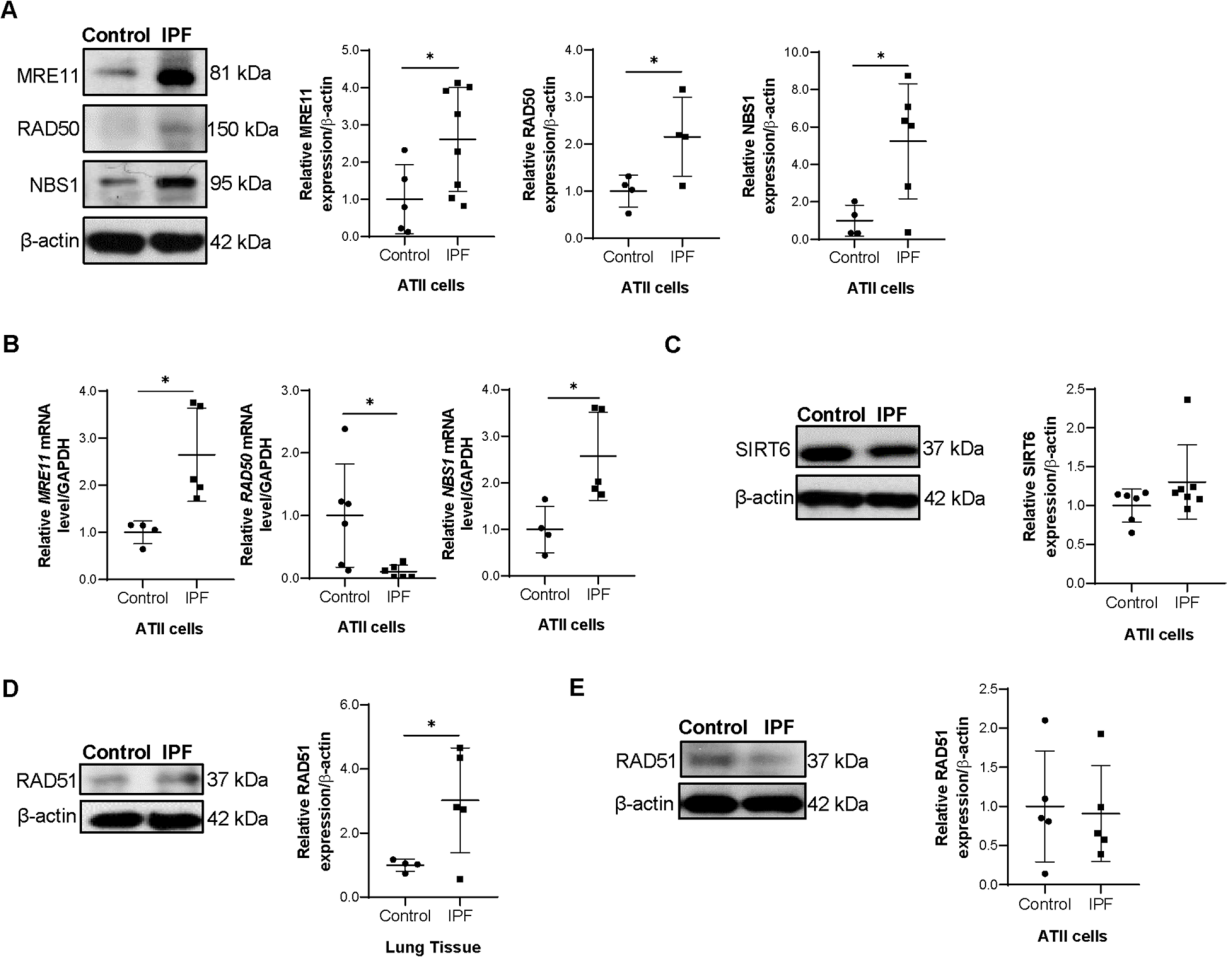
DSB repair in ATII cells in IPF. Lung tissue and ATII cells obtained from control organ donors and IPF patients were used for the analysis. **A** MRE11, RAD50, and NBS1 protein expression in ATII cells was determined by western blotting. The quantification is also shown. **B** *MRE11*, *RAD50*, and *NBS1* gene levels in ATII cells were assessed by RT-PCR. **C** Western blot images and quantification of SIRT6 in ATII cells. RAD51 protein expression was analyzed in lung tissue (**D**) and ATII cells (**E**) by western blotting. Quantifications are also shown. Data are normalized to controls and expressed as means ± SD. *N* = 4–8 lungs per group; *p < 0.05

Since we detected oxidative stress, DNA damage, and an ineffective antioxidant defense system in ATII cells, we wanted to evaluate the capability and mechanism of DDR. Specifically, we investigated whether observed cell alterations in IPF were associated with impaired DDR. There are two types of NHEJ: canonical (c-NHEJ) and alternative (alt-NHEJ) [51]. Early response of the canonical DSB-associated DNA repair genes includes the MRE11-RAD50-NBS1 (MRN) complex [52]. It is among the first factors recruited to DNA breaks, as it senses DSBs and triggers DDR, and plays a role in promoting NHEJ [53]. Our results indicate that the levels of MRE11, RAD50, and NBS1 proteins were significantly upregulated in ATII cells from IPF compared to controls (Fig. 3A). *MRE11* and *NBS1* mRNA expression were upregulated, and *RAD50* levels were downregulated in ATII cells obtained from patients with this disease (Fig. 3B). SIRT6 is a DNA repair factor that contributes to the NHEJ pathway [54]. We haven't observed any differences in its levels in ATII cells between the groups by western blotting, suggesting that it does not promote DSB repair in IPF (Fig. 3C).

DSBs can be repaired through either HR or NHEJ [55]. RAD51 is an evolutionarily conserved recombinase that plays a critical role in the HR repair of DSBs and is recruited to the lesion sites [56, 57]. Our data show its higher expression in lung tissue obtained from IPF compared to controls (Fig. 3D). However, the HR repair pathway wasn't activated in ATII cells isolated from patients with this disease (Fig. 3E). Together, these results suggest the preference for NHEJ in ATII cells for DNA damage repair in IPF.

### Deficient DDR machinery in IPF

**Fig. 4:**
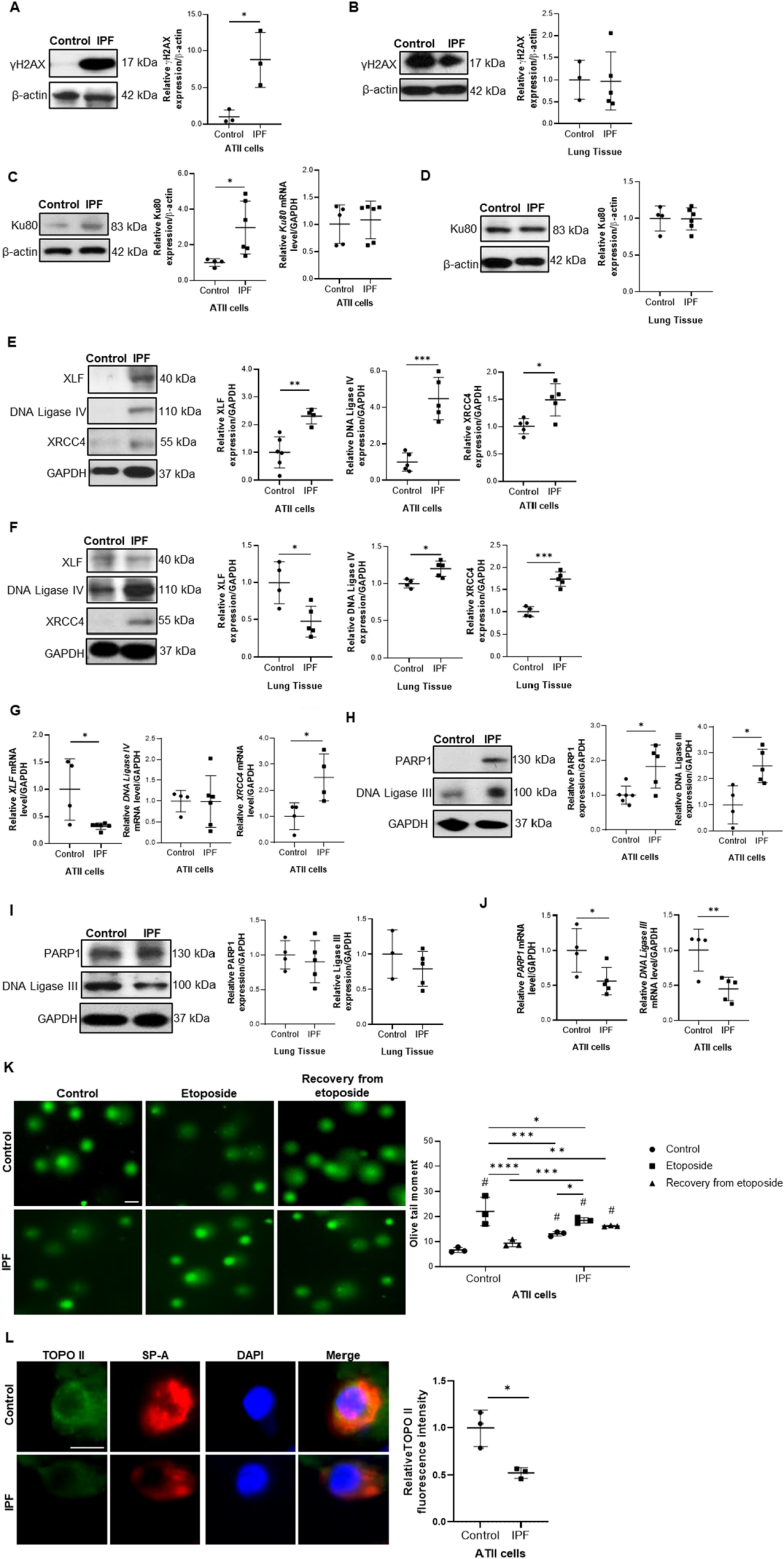
DNA damage response in ATII cells in IPF. Protein and mRNA expression were determined in lung tissue and ATII cells obtained from control organ donors and IPF patients. γH2AX levels in ATII cells (**A**) and lung tissue (**B**) were analyzed by western blotting. Quantifications are also shown. Ku80 protein and mRNA expression and quantifications in ATII cells (**C**) and lung tissue (**D**). XLF, DNA Ligase IV, and XRCC4 protein levels were determined in ATII cells (**E**) and lung tissue (**F**). Densitometric quantifications are also shown. **G ***XLF*, *DNA Ligase IV*, and *XRCC4* mRNA levels in ATII cells. Western blot images and quantification of PARP1 and DNA Ligase III in ATII cells (**H**) and lung tissue (**I**). **J** *PARP1* and *DNA Ligase III *mRNA levels in ATII cells are shown. **K** DNA damage induced by etoposide in ATII cells after 24 h and recovery in a drug-free medium after 24 h were assessed using a comet assay. Olive tail moment was analyzed. **L** The lung sections were stained with TOPO II (green), SP-A (red), and DAPI (blue) by immunofluorescence. TOPO II fluorescence intensity in ATII cells is shown. Data are normalized to controls and expressed as means ± SD. N = 3-6 lungs per group; #p<0.05 compared to control conditions in control organ donors; *p<0.05; **p<0.01; ***p<0.001; ****p<0.0001

An early step in the cellular response to DSBs includes H2AX phosphorylation [58]. Here, we wanted to define the DSB repair capacity in ATII cells in IPF. Increased γH2AX levels were detected in ATII cells from patients compared with control organ donors, indicating activation of the DDR (Fig. 4A). However, DNA damage persisted in these patients. Moreover, no significant difference in γH2AX expression was observed between the analyzed groups in lung tissue (Fig. 4B).

Ku proteins sense broken DNA ends and promote the recruitment of DNA damage repair proteins [59]. Our results show an increase in Ku80 protein expression in ATII cells in IPF and no significant changes at the gene level (Fig. 4C). Additionally, we did not detect any difference in Ku80 protein expression in lung tissue (Fig. 4D). The late stages of NHEJ include the XLF-XRCC4-DNA Ligase IV complex, which is recruited to sites of damage to promote ligation [60]. Significantly higher expressions of all proteins were detected in ATII cells in IPF (Fig. 4E). Also, in lung tissue, DNA Ligase IV and XRCC4 protein levels increased, whereas XLF expression was decreased in this disease (Fig. 4F). *XLF* and *XRCC4* mRNA levels were downregulated and upregulated, respectively in ATII cells in IPF, and no significant changes were detected for *DNA Ligase IV* expression in the analyzed groups (Fig. 4G). Our results indicate an activation of DDR in ATII cells in patients with this disease.

The molecular mechanisms of the alt-NHEJ repair process involve PARP1 and DNA Ligase III. Their levels were analyzed in ATII cells and lung tissue to determine the efficiency of this repair pathway. Their expression was higher in ATII cells from IPF patients than in controls, suggesting the activation (Fig. 4H). However, no significant differences were detected in lung tissue (Fig. 4I). *PARP1* and *DNA Ligase III* mRNA levels were decreased in ATII cells in this disease (Fig. 4J). We detected activation of both c-NHEJ and alt-NHEJ in ATII cells in IPF; however, our results suggest that repair is inefficient.

### ATII cells in IPF have decreased DNA repair capacity

Next, we wanted to define DNA damage and explore further ineffective NHEJ in ATII cells obtained from IPF. Isolated ATII cells were treated with 50 µM etoposide for 24 h. This DNA-damaging agent was used to determine repair capacity by a comet assay (Fig. 4K). Olive tail moments were higher in ATII cells isolated from IPF patients than from control organ donors. Etoposide-induced DNA damage was assessed in ATII cells obtained from both groups, and was decreased in IPF. The recovery was determined after 24 h in drug-free media, and higher Olive tail moments were observed in ATII cells from IPF compared with controls. These results indicate that ATII cells from patients with this disease have reduced capacity to repair damaged DNA. Etoposide is the primary molecular target of topoisomerase II (TOPO II) [61]. Therefore, we assessed its levels in ATII cells obtained from controls and IPF patients. Our results show that TOPO II expression was significantly reduced in this disease (Fig. 4L). Etoposide requires TOPO II to generate DSBs and its lower levels may explain the diminished Olive tail moments observed in ATII cells in IPF following this treatment compared with control organ donors. Together, our results show that ATII cells from IPF patients exhibit impaired DNA repair capacity, leading to persistently elevated DNA damage following etoposide exposure.

### Co-localization of DNA damage-repair proteins with dynein

**Fig. 5:**
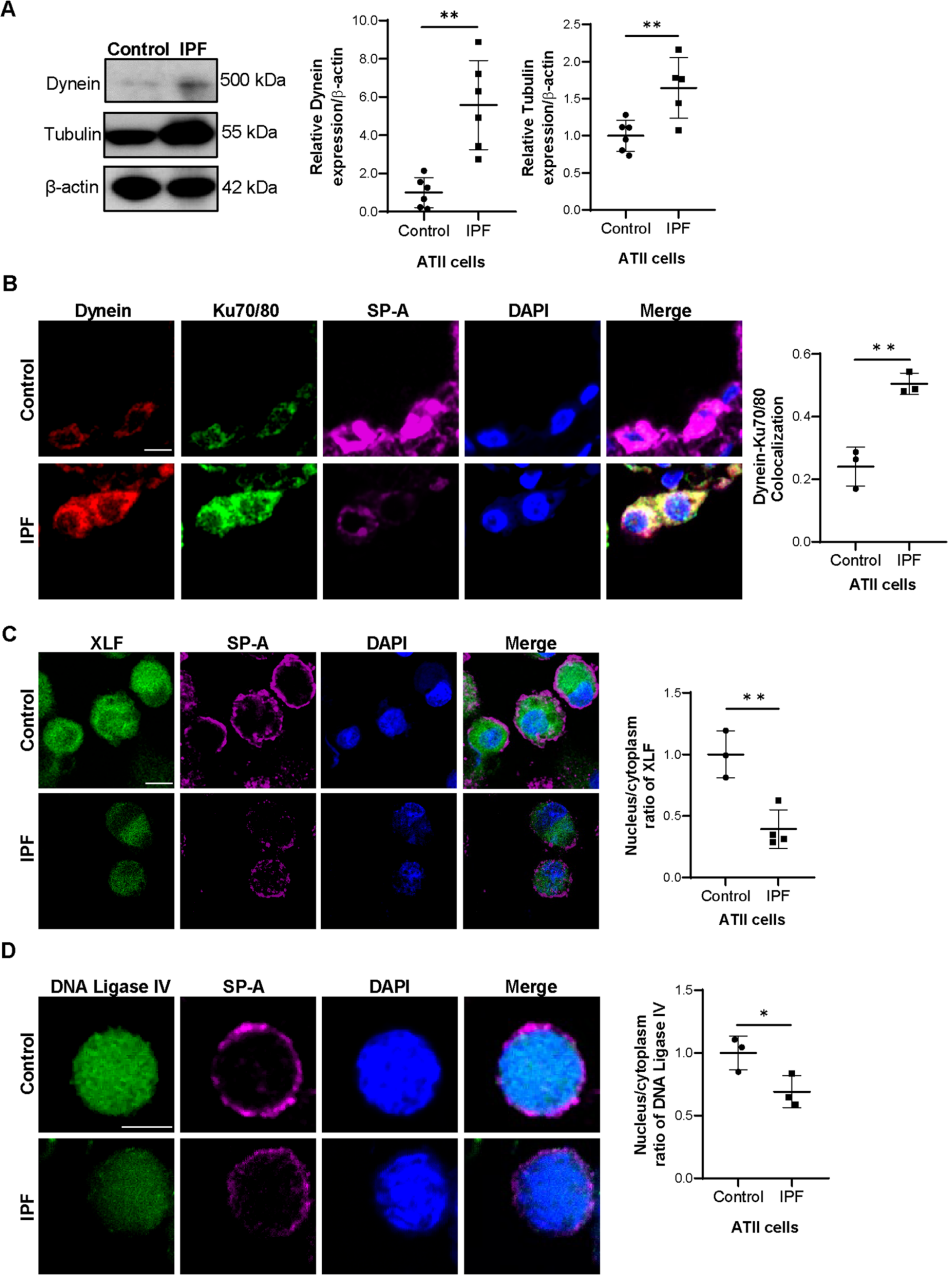
Localization of DNA damage repair proteins in ATII cells in IPF. Lung tissue and ATII cells were obtained from control organ donors and IPF patients. **A** Dynein and tubulin levels were examined by western blotting in ATII cells, and densitometric quantifications were shown. **B** Human lung sections were stained using dynein (red), Ku70/80 (green), and SP-A (magenta) antibodies, and DAPI (blue) by immunofluorescence. Dynein and Ku70/80 co-localization was analyzed, and Pearson's correlation coefficient is shown. ATII cell cytospins were stained with XLF (green) (**C**), DNA Ligase IV (green) (**D**), SP-A (magenta), and DAPI (blue) by immunofluorescence. The fluorescence intensity was analyzed using ImageJ and shown as the nucleus/cytoplasm ratio. Data are normalized to controls and expressed as means ± SD (scale bar—5 µm). *N* = 3–6 lungs per group; *p < 0.05; **p < 0.001

Microtubules (MTs) can regulate genome organization and stability by exerting mechanical forces on the nucleus [62]. Also, they play a role in trafficking DNA damage repair proteins to the nucleus, including the MT motor protein dynein [63]. Since proteins involved in DNA repair are associated with dynein and MTs, disrupting their trafficking can exacerbate DNA damage. Dynein and tubulin expression were analyzed by western blotting in ATII cells isolated from IPF and control organ donors. Interestingly, we found that their expression was higher in this disease than in controls (Fig. 5A). Next, we analyzed whether NHEJ repair proteins interact with MTs and translocate into the nucleus. Ku70/80 co-localization with dynein was increased in ATII cells from IPF patients (Fig. 5B). We aimed to determine whether this interaction enhances the trafficking of DNA damage and repair proteins to the nucleus to repair DSBs. DDR proteins, such as XLF and DNA ligase IV, were sequestered in the cytoplasm (Fig. 5C, D). These results suggest that loading DNA damage and repair proteins onto MTs did not enhance their nuclear trafficking, thereby contributing to inefficient DNA damage repair.

### Alterations of nuclear mechanical properties in ATII cells in IPF

**Fig. 6:**
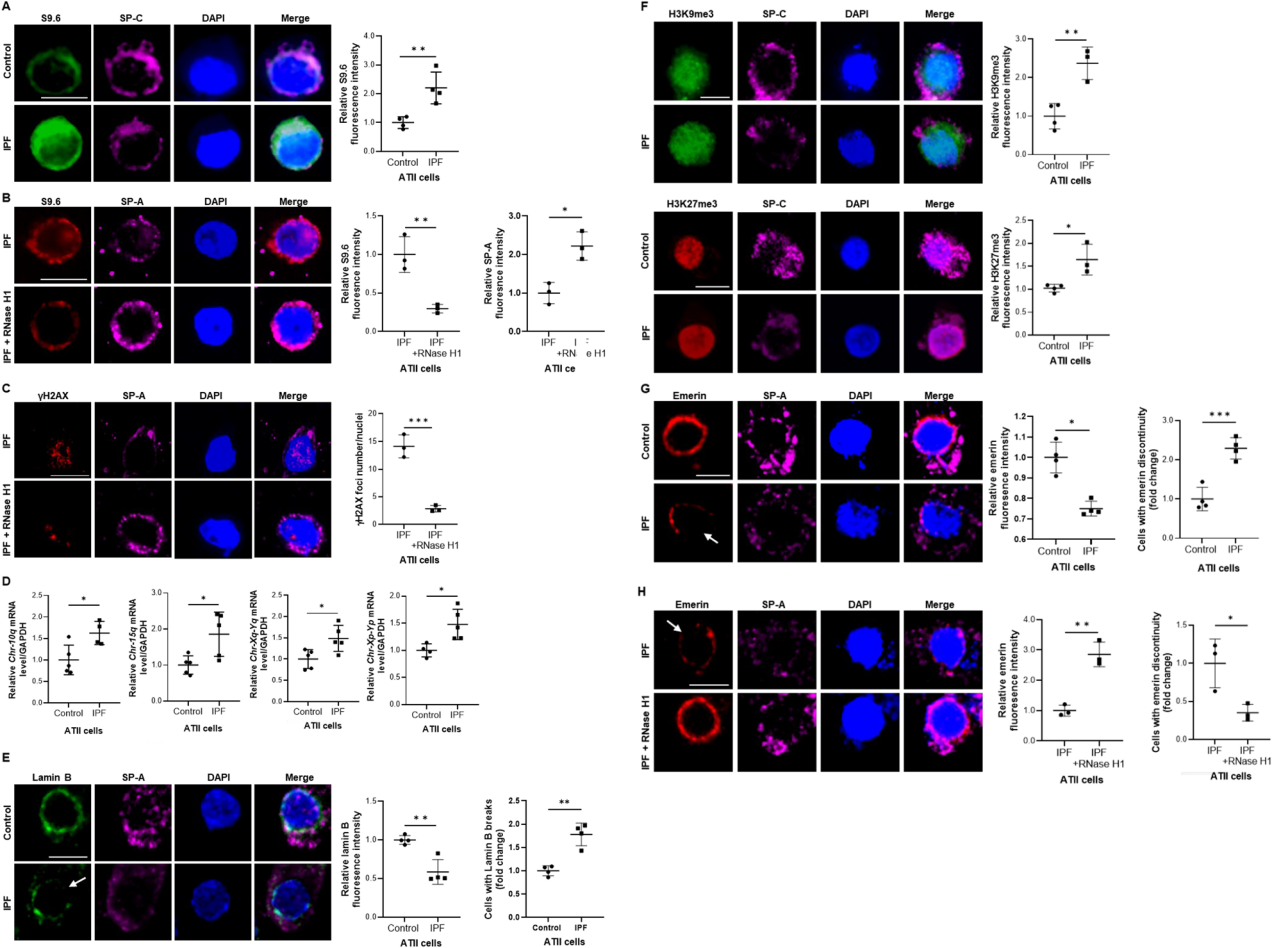
Alteration of genetic and epigenetic markers in ATII cells in IPF. Lung tissue and ATII cells were obtained from control organ donors and IPF patients. **A** The staining was performed on ATII cell cytospins using S9.6 (green) and SP-C (magenta) antibodies by immunofluorescence (DAPI, blue). The S9.6 fluorescence intensity is shown. **B, C** Freshly isolated ATII cells from IPF patients were transfected with the RNase H1 vector and analyzed by immunofluorescence. They were stained with S9.6 (red) or γH2AX (red), SP-A (magenta), and DAPI (blue). The relative fluorescence intensity of S9.6 and SP-A is shown, and the number of γH2AX foci was quantified. **D** The chromosome 10q, 15q, Xq-Yq, and Xp-Yp levels in ATII cells were determined by qPCR. **E** ATII cell cytospins were stained with lamin B (green) and SP-A (magenta) antibodies and DAPI (blue) by immunofluorescence. The lamin B fluorescence intensity and breaks were quantified. **F** ATII cell cytospins were stained for H3K9me3 (green) or H3K27me3 (red), SP-C (magenta), and DAPI (blue) by immunofluorescence. Their fluorescence intensity is shown. **G** ATII cell cytospins were stained with emerin (red) and SP-A (magenta) antibodies and DAPI (blue) by immunofluorescence. Emerin fluorescence intensity and the cells with emerin discontinuity were analyzed. **H** ATII cells from IPF were transfected with the RNase H1 vector and stained using emerin (red), SP-A (magenta), and DAPI (blue). The emerin fluorescence intensity and cells with emerin discontinuity were analyzed (scale bar: 5µm). N = 3 - 5 lungs per group; *p<0.05; **p<0.01. ***p<0.001

MT‐based forces can affect nuclear stiffness and morphology, thereby altering chromatin structure and inducing DNA reorganization. Indeed, previous studies have shown that mechanical tension and stretching can lead to DNA damage [64, 65]. Moreover, ATII cells in the IPF microenvironment are subjected to elevated mechanical tension and pathological stretch [10]. We found high α-SMA expression in lung tissue from IPF (Fig. S2). This suggests an increase in myofibroblasts, which can impact ATII cell function [66]. R-loops (RNA-loops) are nucleic acid structures that form when an RNA strand invades DNA [67–69]. Increasing evidence implicates their abnormal accumulation as a key driver of genomic instability [70]. We determined whether R-loops are associated with nuclear lamina organization and stiffness. The S9.6 antibody recognizes the DNA:RNA structure [68]. Increased formation of R-loops was observed in ATII cells from IPF patients compared with controls by immunofluorescence (Fig. 6A). These data suggest that their abnormal accumulation may contribute to and exacerbate DNA damage. To confirm this, we performed rescue experiments to resolve R-loops by transfecting primary ATII cells isolated from IPF patients with RNase H1 and culturing the cells for 48 h. As shown in Fig. 6B, RNase H1 overexpression markedly reduced S9.6 levels compared with the empty vector control, indicating efficient resolution of R-loops. Notably, we also detected increased SP-A fluorescence intensity in ATII cells overexpressing RNase H1 and a decreased γH2AX foci, demonstrating attenuation of DNA damage (Fig. 6C). These results provide direct functional evidence that R-loop accumulation is a causal driver of DNA damage in ATII cells in IPF, and that resolving R-loops can reduce these pathological features.

TERRA is the long noncoding RNA (lncRNA) telomeric repeat-containing RNA, and its excess can lead to impaired telomere replication and genomic instability [71]. R-loops at telomeres are analyzed at specific chromosome ends by qPCR [72]. Telomeric R-loops were detected in four chromosomes: 10q, 15q, Xp-Yp and Xq-Yq in ATII cells. TERRA transcripts were higher in ATII cells obtained from IPF (Fig. 6D). Together, these data suggest aberrantly increased R-loops levels in ATII cells in patients, which can contribute to replication stress, transcription replication conflicts, and genomic instability.

To investigate the adaptation of nuclear architecture to microenvironmental forces in IPF, we assessed lamin B protein levels. It is coupled with nuclear envelope stability and lamina organization [65, 73]. Interestingly, a significant increase in the number of lamin B breaks and a decrease in its expression were detected in ATII cells from IPF patients compared with control organ donors by immunofluorescence (Fig. 6E). Next, we sought to determine whether microenvironment-derived forces and elevated TERRA levels in ATII cells in IPF affect the heterochromatic state. Two major epigenetic histone repressive methylations, H3K9me3 and H3K27me3, are involved in gene regulation [74]. Their increased expression in ATII cells in disease compared with controls was detected by immunofluorescence (Fig. 6F). Our results suggest that nuclear mechanical stress increases heterochromatin levels and alters the chromatin state, contributing to DNA damage. The observed low lamin B levels in ATII cells in IPF may affect nuclear membrane integrity. We wanted to further determine whether the increased mechanical stress contributes to the increased tension in the nuclear envelope. ATII cells obtained from patients exhibited compromised nuclear morphology, characterized by shape alterations and discontinuities in emerin, an inner nuclear membrane protein (Fig. 6G). Reduced emerin levels and an increased number of cells with emerin discontinuity at the nuclear lamina were detected in ATII cells from IPF using immunofluorescence. This suggests a failed repair, which can lead to nuclear envelope rupture. To further assess the impact of R-loops on nuclear envelope stress, ATII cells isolated from patients with this disease were transfected with the RNase H1 vector as described above. Importantly, RNase H1 overexpression significantly increased emerin expression and reduced its discontinuity in cells, a causal driver of nuclear envelope perturbation, compared to untreated ATII cells from IPF (Fig. 6H). Together, our data indicate that external compression forces elevated heterochromatin levels and led to abnormal R-loop accumulation, which, in turn, induce genomic instability in this disease. We provided direct functional evidence that resolving R-loops reduces stress on nuclear envelopes.

### Mechanical tension alters ATII cell chromatin function and nuclear structure

**Fig. 7:**
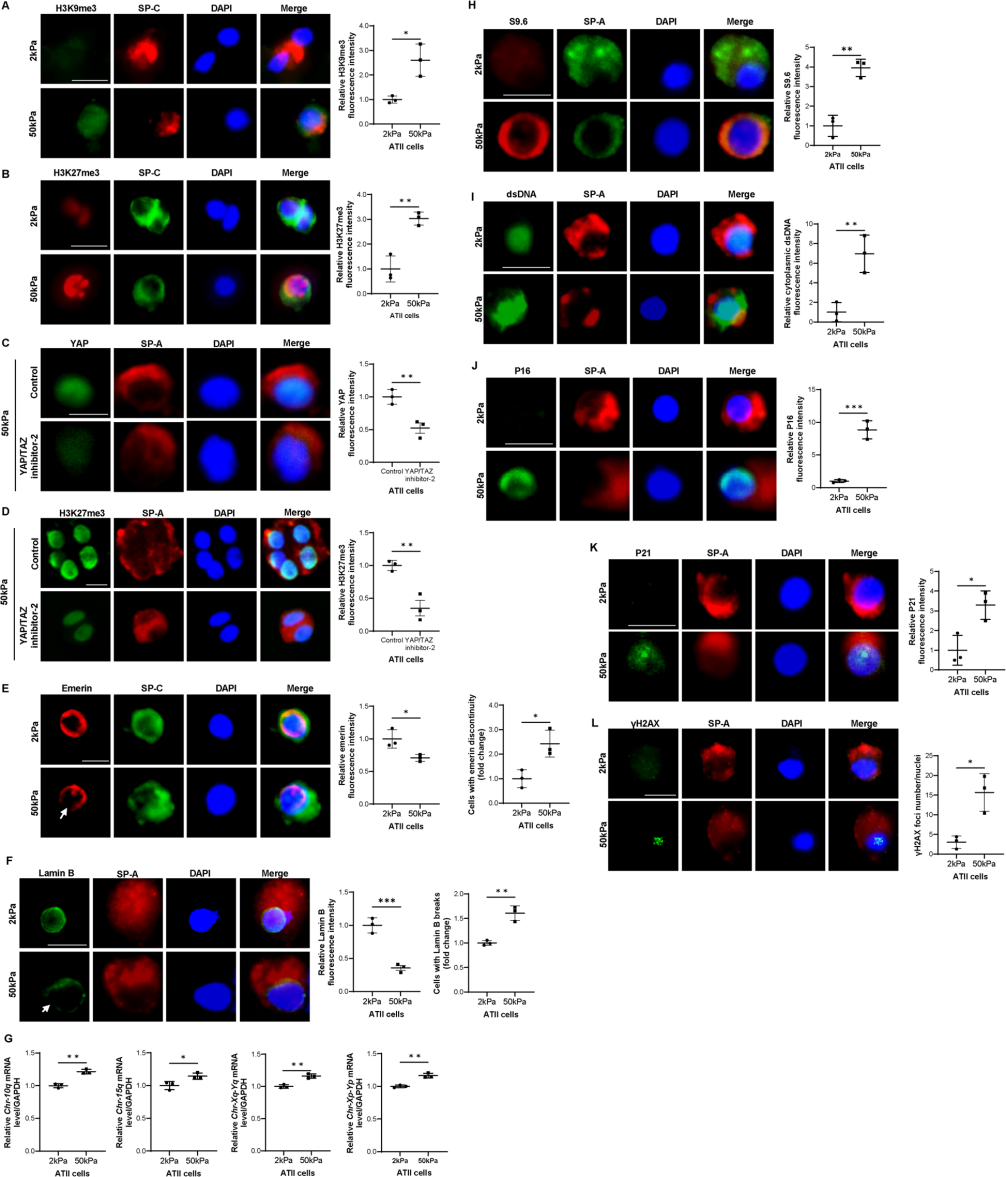
Mechanical stiffness alters ATII cell function. ATII cells were isolated from control organ donors, cultured on 2 kPa and 50 kPa PDMS hydrogel for 48 h, and analyzed using immunofluorescence. Cells were stained with SP-C, DAPI, and H3K9me3 (**A**) or H3K27me3 (**B**). Their relative fluorescence intensity is shown. ATII cells were treated with YAP/TAZ inhibitor-2 and cultured on 50 kPa PDMS hydrogel for 48 h. **C** YAP (green) or (**D**) H3K27me3 (green) antibodies with SP-A (red), and DAPI (blue) were used by immunofluorescence. Their relative fluorescence intensity is shown. **E** Emerin (red), SP-C (green), and DAPI (blue) staining was performed in ATII cells. Emerin fluorescence intensity and cells with emerin discontinuity were analyzed. **F** Lamin B (green), SP-A (red), and DAPI (blue) were used for immunofluorescence in ATII cells. **G** TERRA transcripts from chromosomes 10q, 15q, Xq-Yq, and Xp-Yp in ATII cells were detected by qPCR. **H** Expression of S9.6 (red), (**I**) cytoplasmic dsDNA (green), (**J**) P16 (green), (**K**) P21 (green**)**, and (**L**) γH2AX (green) in ATII cells by immunofluorescence (DAPI-blue, SP-A-green or red). Relative fluorescence intensity is shown (scale bar—10 µm). *N* = 3 lungs; *p < 0.05; **p < 0.01; ***p < 0.001

We determined the impact of increased mechanical stiffness on ATII cells. A PDMS hydrogel mimics physiological tension [41]. ATII cells isolated from control organ donors were cultured on 2 kPa and 50 kPa PDMS substrates for 24 h and 48 h. Expression of H3K9me3 and H3K27me3 was assessed by immunofluorescence. Their levels were higher in ATII cells cultured on a 50 kPa hydrogel than on a 2 kPa hydrogel for 48 h (Fig. 7A, B). Additionally, after 24 h of culture, H3K27me3 levels were significantly elevated, and a trend toward increased H3K9me3 expression was observed (Fig. S3A, S3B).

Since YAP activity is regulated by ECM stiffness [75], we analyzed the impact of its inhibition on ATII cells obtained from control organ donors and cultured on a 50 kPa PDMS hydrogel. Our results indicate that cell treatment with 2.5 nM YAP/TAZ inhibitor-2 for 48 h significantly reduced YAP levels compared with untreated ATII cells, as assessed by immunofluorescence (Fig. 7C). Next, we assessed H3K27me3 expression following this treatment. Stiffness inhibition by YAP/TAZ inhibitor-2 reduced its levels in ATII cells (Fig. 7D), demonstrating that mechanotransduction signaling directly influences epigenetic regulation in response to mechanical forces. We further analyzed the impact of stiffness on emerin expression in ATII cells. Its levels were significantly decreased, and discontinuity increased after 48 h of ATII cell culture on 50 kPa PDMS hydrogels compared with 2 kPa (Fig. 7E). We didn’t observe any changes after 24 h (Fig. S3C). Lamin B is another marker of nuclear membrane integrity [76]. ATII cells cultured on stiff 50 kPa PDMS hydrogels exhibited a significant reduction in lamin B intensity and a marked increase in breaks compared with cells cultured on soft 2 kPa substrates (Fig. 7F). This suggests that increased matrix stiffness is sufficient to induce nuclear envelope destabilization and nuclear damage–associated ATII cell phenotypes.

Here, we aimed to investigate alterations in control ATII cells subjected to mechanical stiffness. We found higher TERRA transcript levels on chromosomes 10q, 15q, Xq-Yq, and Xp-Yp in ATII cells cultured for 48 h on 50 kPa compared with 2 kPa PDMS hydrogels (Fig. 7G). Moreover, in ATII cells cultured on 50 kPa hydrogels, we detected increased DNA-RNA hybrid R-loop formation after 48 h, as indicated by higher S9.6 expression (Fig. 7H). Additionally, an accumulation of cytoplasmic dsDNA (Fig. 7I), increased P16 (Fig. 7J) and P21 expression (Fig. 7K), and γH2AX foci (Fig. 7L) were detected in these culture conditions. In addition, mRNA levels of key components of the DNA repair and antioxidant pathways were assessed. As shown in Fig. S4, substrate stiffness was associated with significant alterations in *PARP1*, *DNA Ligase IV*, *DJ-1*, *XRCC4*, *RAD50,* and *MRE11* expression. Together, our data indicate that ATII cells in IPF experience external compression forces, leading to nuclear envelope alterations, elevated heterochromatin levels, and abnormal R-loop accumulation, which, in turn, induce genomic instability and senescence. These results indicate that external mechanical forces are a mechanism underlying ATII cell dysfunction in this disease.

## Discussion

It has been reported that ATII cells drive fibrosis in IPF [77]. However, the relationships between nuclear stiffness, mechanical stress, and DNA damage are poorly understood, and studies using ATII cells isolated from patients with this disease are limited. Here, we found that the mechanical cellular stress in IPF contributes to sustained DNA damage and activates DDR. ATII cells exhibited lower lamin B levels, increased R-loops, and nuclear envelope discontinuity, indicating nuclear alterations.

DSBs and accumulating unrepaired DNA lesions drive various diseases via the induction of genomic rearrangements, cellular senescence, or apoptosis [78–80]. They are observed in response to DNA damage and depend on the severity of the damage, the stimuli, and the cell type. The tumor suppressor *P53* is a known key regulator of senescence, promotes cell cycle arrest and activates the downstream target *P21* [81]. Our results showed that the P53/P21 pathway is upregulated in ATII cells in IPF and correlated with DNA damage. We also detected increased β-galactosidase activity and P16 expression, markers of cell senescence.

ATII cells obtained from IPF had upregulated NHEJ, indicating activation of the DDR to repair DSBs. We used a comet assay to further define DNA damage. ATII cells isolated from these patients and control organ donors had similar levels of Olive tail moments after treatment with etoposide. However, after recovery in media without drug, ATII cells obtained from IPF had higher DNA damage. These results suggest a lower repair capacity, which can contribute to cell senescence. Maintaining the genome's integrity is vital to all cells [82, 83]. Our results suggest that the failure of DNA repair mechanisms compromises nuclear integrity in IPF. Also, they indicate that NHEJ is a hallmark of ATII cells obtained from patients with this disease, and this unfaithful pathway can contribute to genomic instability.

We found sustained higher γH2AX levels in ATII cells from IPF, indicating increased DNA damage signaling. RAD51 promotes the survival of replication stress and prevents the accumulation of replication-associated DSBs [84]. However, its protein levels were unchanged in ATII cells from IPF compared with controls, indicating inactivation of the HR pathway. Our results suggest that ATII cells promote NHEJ as the main DSB repair pathway in this disease. Indeed, we found canonical and alternative NHEJ activation, revealing their role in IPF. It has been shown that DDR also influence microtubule stability and dynamics, and connect DNA insults to the cytoskeleton [85]. In agreement with this study, we observed a significant increase in the levels of DDR factors, dynein, and tubulin proteins in ATII cells obtained from patients with IPF compared with controls. This demonstrates that DNA damage repair is inefficient in this disease. During NHEJ, the Ku70/80 complex rapidly binds to DNA ends in response to DSBs; after that DNA-PKcs, followed by the recruitment of other factors, which subsequently generate ligatable ends [25, 86].

We detected fewer ATII cells in IPF lung tissue. Studies showed that ATII cells have impaired differentiation into ATI cells, leading to alveolar wall damage in pulmonary fibrosis [4, 87]. Our previous results indicate that ATII cell dysfunction plays a critical role in the pathogenesis of IPF [12]. Here, we observed that persistent DNA damage and defective DNA repair are crucial for disease development. Notably, the cell cycle can be blocked by unresolved DNA damage [88]. Our results indicate that ATII cells isolated from IPF patients exhibit increased DDR expression and have impaired NHEJ. Multiple mechanisms can account for impaired DNA damage repair in ATII cells. We found higher ROS levels in ATII cells from these patients. Elevated ROS can damage multiple macromolecules, including lipids and proteins, and induce DNA base oxidation, SSBs (single-strand breaks), and DSBs [89]. They can also alter cell proliferation and differentiation [90]. Moreover, alterations in telomere maintenance and surfactant production have been associated with the development of IPF [91, 92]. Our data suggest that ATII cell loss via inefficient NHEJ and increased DNA damage represents a novel molecular mechanism contributing to this disease.

We show that maladaptive mechanical stress in IPF leads to persistent DNA damage signaling, associated with deficient DNA repair. This can affect gene expression and other cellular processes [93, 94]. Recent studies have shown that mechanical stress can induce nuclear envelope rupture and uncontrolled exchange of nucleocytoplasmic contents, leading to DNA damage [95, 96]. Moreover, dysfunctional ATII cells in IPF are typically located adjacent to fibroblastic foci, thereby resulting in sustained exposure to elevated mechanical tension [97]. This constant mechanical stress can impact genome integrity [98]. External forces and increased expression of dynein and microtubules lead to cytoskeletal stiffness [99]. This can impair the intracellular trafficking of DNA repair proteins in ATII cells in the IPF microenvironment. Therefore, the observed increased ROS levels, mechanical stress, and DNA damage may further contribute to ATII cell senescence in IPF.

Recent work by Nava et al. suggests that microenvironment-derived forces can deform and remodel chromatin, altering gene expression [100]. Additionally, TERRA is associated with heterochromatin states at telomeres [101, 102]. R-loops are an RNA–DNA hybrid formed between a nascent transcript and the template DNA strand and can threaten genome integrity [68]. There is a strong association between R-loops and DNA damage and diseases [67–69, 103]. While R-loops are recognized as potential sources of genotoxic stress, little is known about the physiological events that drive their formation in pulmonary pathology, particularly in ATII cells in IPF. Aberrant R-loops formation can impair transcription elongation, leading to replication stress, DNA breaks, and genomic instability [104]. Our data suggest that R-loop accumulation is the primary driver of DNA damage in ATII cells in IPF. This is evidenced by the decrease in γH2AX foci following RNase H1 overexpression, which eliminates R-loops. Lamin B plays an essential role in nuclear chromatin organization, primarily by anchoring heterochromatin territories to the nuclear membrane [73, 105]. It has been reported that cells defective in lamin B are sensitive to compression forces, leading to nuclear membrane rupture [106]. Therefore, the observed decrease in lamin B expression in ATII cells in IPF could be associated with impaired heterochromatin chain fluctuations and nuclear displacement. Our results indicate impaired nuclear responses to mechanical tension and persistent stress at the nuclear membrane. This is in agreement with a study showing nuclear deformations associated with the selective loss of lamin B [107]. Also, H3K27me3 and H3K9me3 are histone marks specific for constitutive and facultative heterochromatin [108]. High levels of heterochromatin and lamin B depletion may affect nuclear stiffness and envelope integrity [100, 109]. Increasing levels of H3K27me3 and H3K9me3 in ATII cells in IPF can indicate repressed transcriptional activity in neighboring genome regions. Indeed, it has been reported that nuclear deformation leads to chromatin remodeling mediated by H3K9me3 and H3K27me3 [100]. Also, microtubules and dynein apply forces on the nucleus [99]. We found increased levels of dynein and tubulin in ATII cells from patients with IPF compared with controls. This suggests their contribution to nuclear membrane deformation and shape alterations, and envelope breakdown [102, 103]. It may also explain the observed emerin discontinuity and decreased expression in ATII cells from IPF.

Furthermore, we examined the impact of increased stiffness on control ATII cell function using a PDMS hydrogel system. A significant increase in H3K9me3 and H3K27me3 levels was detected in ATII cells cultured on 50 kPa PDMS hydrogel. Consistent with this, using the YAP/TAZ inhibitor-2, we observed a decrease in H3K27me3 in control ATII cells. These findings show how stiffness regulates epigenetic control of chromatin structure, aligning with our results using freshly isolated ATII cells from IPF. Observed decreased emerin expression and a higher number of cells with emerin discontinuity in cultured ATII cells on 50 kPa PDMS hydrogel may suggest DNA leakage, damage, and instability. Moreover, induced mechanical stiffness in control ATII cells led to increased R-loop formation, elevated TERRA levels, cytoplasmic dsDNA leakage, and increased γH2AX foci, P16, and P21 levels. We previously reported that TERRA accumulates at the ends of dysfunctional telomeres in ATII cells obtained from smokers, leading to genome instability [110]. Together, our results suggest that stiffness affects ATII cell function in IPF, potentially contributing to its severity.

## Conclusions

**Fig. 8:**
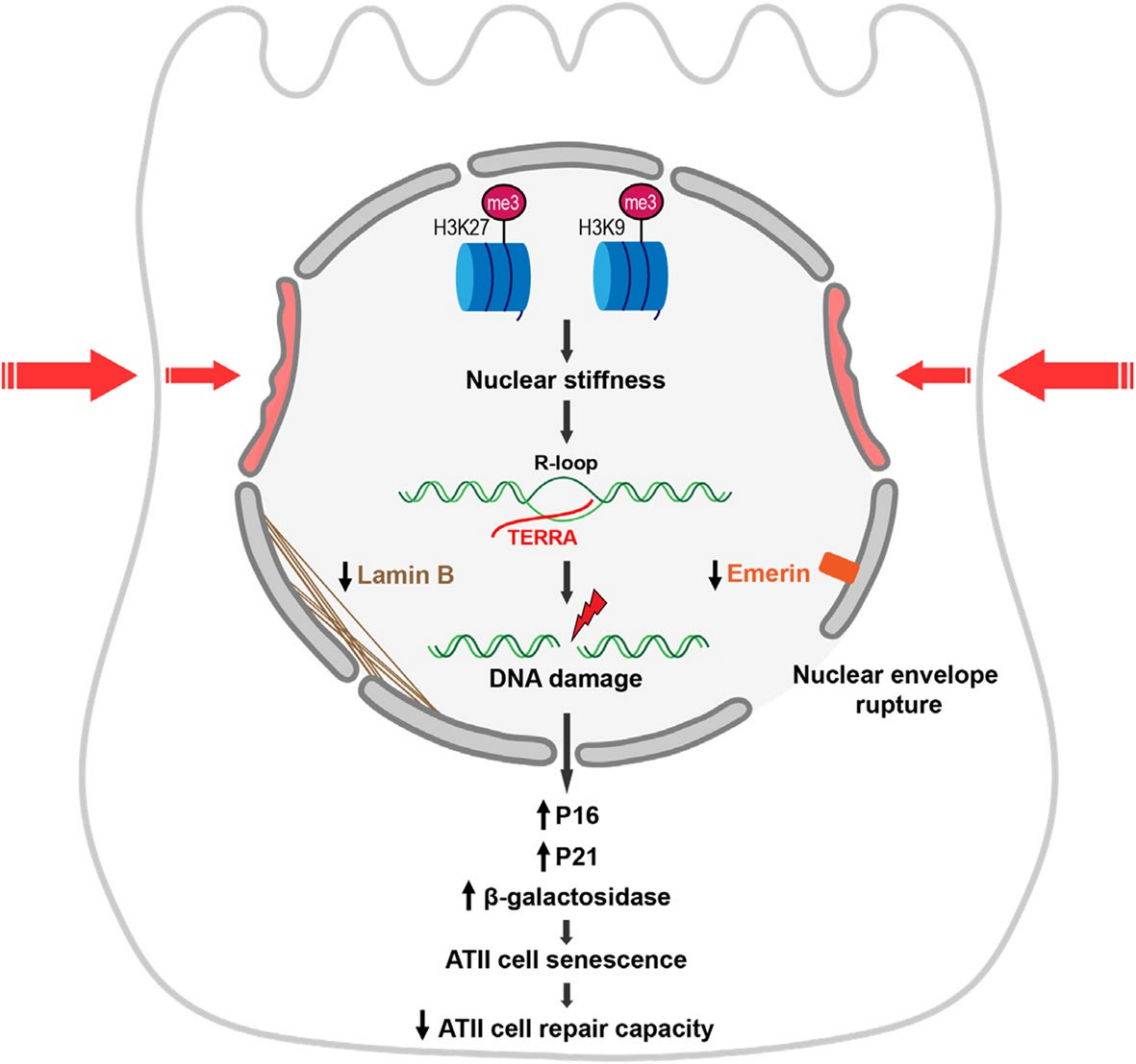
Model of the ATII cell dysfunction in IPF. ATII cells in IPF are exposed to nuclear tensions due to the mechanical stiffness in the microenvironment. High R-loop levels, DNA damage, emerin discontinuity, and low lamin B expression contribute to ATII cell senescence and dysfunction

Here, we demonstrated sustained DNA damage in ATII cells isolated from IPF patients, with nuclear stiffness arising from unphysiological, prolonged mechanical forces associated with the disease's pathophysiology (Fig. 8). Our findings indicate that ATII cells exhibit impaired NHEJ, which may be linked to elevated mechanical tension. Ultimately, mechanical stress in the IPF microenvironment and R-loop-mediated genomic instability can impair alveolar structural and functional homeostasis, thereby accelerating disease progression. Studies of the mechanisms that impair DSB repair are crucial for identifying novel treatment targets. Preventing the pro-senescent effects of mechanical stress and preserving DNA integrity and quality control in ATII cells are promising therapeutic approaches.

## Supplementary Information

Supplementary Material 1: Fig. S1. Control IgG staining. Fig. S2. High α-SMA expression in IPF. Fig. S3. Impact of mechanical stiffness on ATII cells. Fig. S4. mRNA expression of DNA damage-related genes in mechanical stiffness on ATII cells.

Supplementary Material 2: Table. S1. Sequences of primers used for RT-PCR.

## Data Availability

The data generated in the study are included and are available from the corresponding author upon reasonable request.

## Abbreviations

α-SMA: Alpha smooth muscle actin
γH2AX: Gamma H2A histone family member X
4-HNE: 4-Hydroxynonenal
ATII cell: Alveolar type II cell
ATM: Ataxia-Telangiectasia Mutated
ATR: Ataxia-Telangiectasia and Rad3-related
DCF-DA: 2’- 7’-Dichlorodihydrofluorescein Diacetate
DDR: DNA damage response
DNA-PKcs: DNA-dependent protein kinase catalytic subunit
DSBs: Double-strand breaks
H3K9me3: Tri-methyl-histone H3 (Lys9)
H3K27me3: Tri-methyl-histone H3 (Lys27)
IPF: Idiopathic pulmonary fibrosis
lncRNA: Long noncoding RNA
MRE11: Meiotic recombination 11
NHEJ: Non-homologous end-joining
PDMS: Polydimethylsiloxane
qPCR: Quantitative PCR
R-loops: RNA loops
ROS: Reactive oxygen species
RT-PCR: Reverse Transcription Polymerase Chain Reaction
SP-A: Surfactant protein A
SP-C: Surfactant protein C
SRX: Sulfiredoxin
SSBs: Single-strand breaks
TAZ: Transcriptional coactivator with a PDZ-binding motif
TDP1: Tyrosyl-DNA phosphodiesterase 1
TERRA: Telomeric Repeat-containing RNA
TOPO II: Topoisomerase II
XLF: XRCC4-like factor
XRCC4: X-ray repair cross-complementing protein 4
YAP: Yes-associated protein 1
