# Supplementary material for: Stiffness-dependent alveolar type II cell senescence in idiopathic pulmonary fibrosis
Source: Cell Commun Signal. 2026 Apr 25;24:345. doi: 10.1186/s12964-026-02881-5 (PMC13248465; doi:10.1186/s12964-026-02881-5)
Supplement: Supplementary file 1 — Supplementary Material 1: Fig. S1. Control IgG staining. Fig. S2. High α-SMA expression in IPF. Fig. S3. Impact of mechanical stiffness on ATII cells. Fig. S4. mRNA expression of DNA damage-related genes in mechanical stiffness on ATII cells. [file 12964_2026_2881_MOESM1_ESM.docx]

**SUPPLEMENTARY RESULTS**

**Stiffness-dependent alveolar type II cell senescence in idiopathic pulmonary fibrosis**

Chih-Ru Lin^1,2^, Khanutsanan Woranam^2,3^, Hassan Hayek^2,3^, Jonathan Jeger^2^, Loukmane Karim^2^, Beata Kosmider^2,3^, Rafal Kaminski^3,4^, Christopher W. Schultz^5^, Sudhir Bolla^6^, Nathaniel Marchetti^6^, Gerard J. Criner^6^, Karim Bahmed^2,3*^

^1^Department of Biochemistry, School of Medicine, College of Medicine, Kaohsiung Medical University, Kaohsiung, Taiwan

^2^Center for Inflammation and Lung Research, Lewis Katz School of Medicine, Temple University, Philadelphia, PA 19140, USA

^3^Department of Microbiology, Immunology, and Inflammation, Lewis Katz School of Medicine, Temple University, Philadelphia, PA 19140, USA

^4^Center for Neurovirology and Gene Editing, Lewis Katz School of Medicine, Temple University, Philadelphia, PA 19140, USA

^5^Department of Cancer and Cellular Biology, Lewis Katz School of Medicine, Temple University, Philadelphia, PA 19140, USA

^6^Department of Thoracic Medicine and Surgery, Lewis Katz School of Medicine, Temple University, Philadelphia, PA 19140, USA

*Corresponding author: Karim Bahmed, Ph.D.

Department of Microbiology, Immunology, and Inflammation

Center for Inflammation and Lung Research

Temple University

3500 N. Broad Street, Philadelphia, PA 19140

E-mail: [karim.bahmed@temple.edu](mailto:karim.bahmed@temple.edu)

| **Mouse IgG**  **Rabbit IgG**  **DAPI**  **Merge**  **Mouse Antibody**  **Rabbit Antibody**  **DAPI**  **Merge** 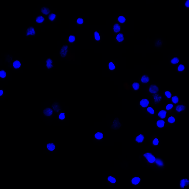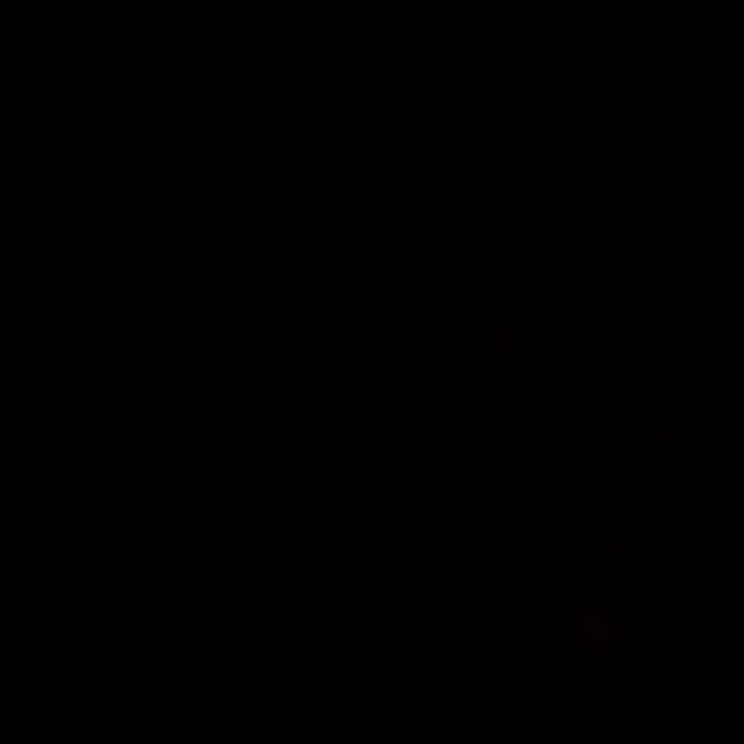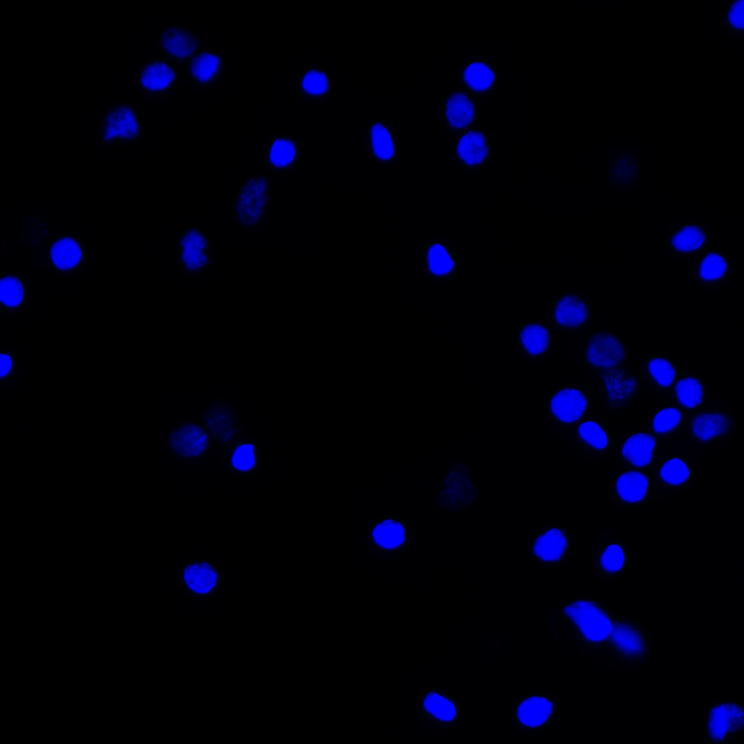 **Goat IgG**  **DAPI**  **Merge** 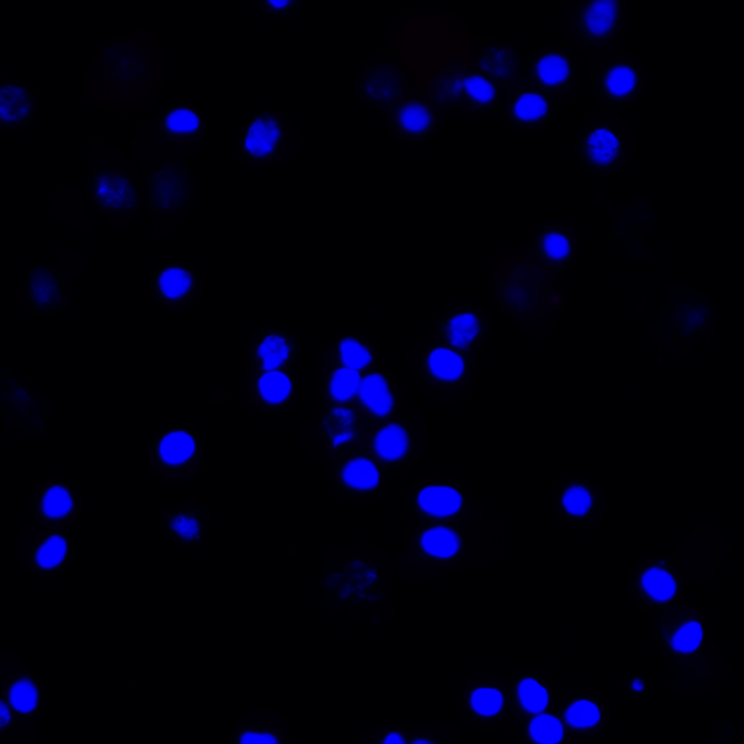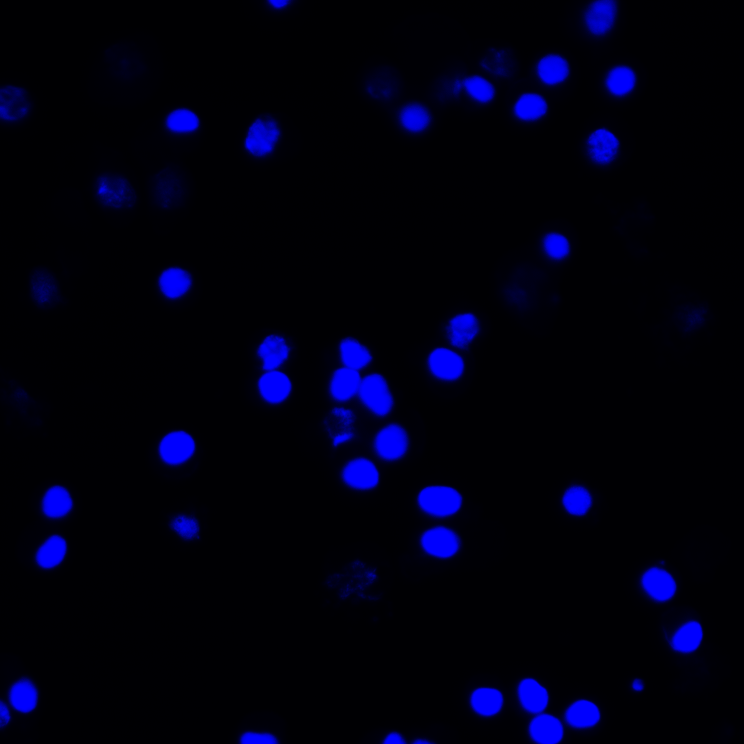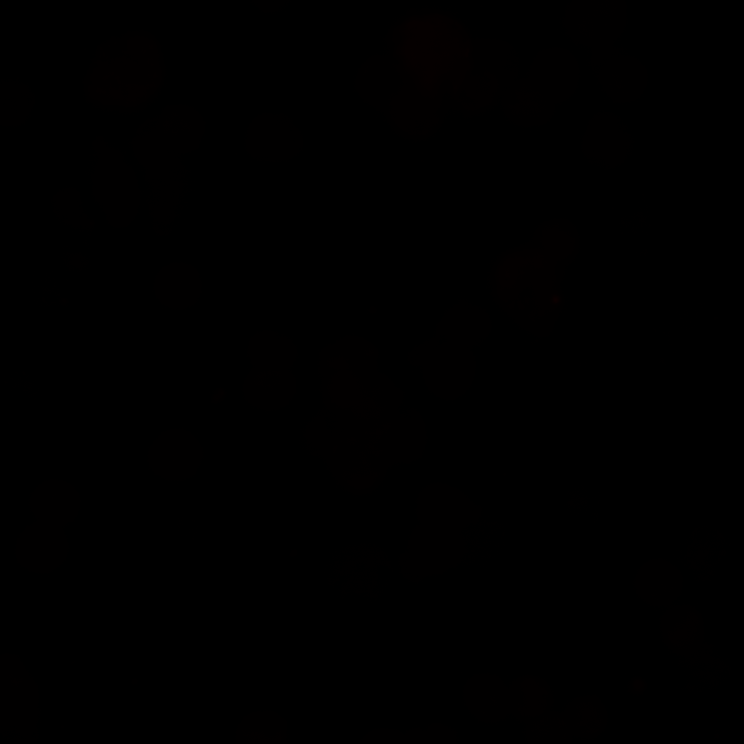 **Goat Antibody**  **DAPI**  **Merge** 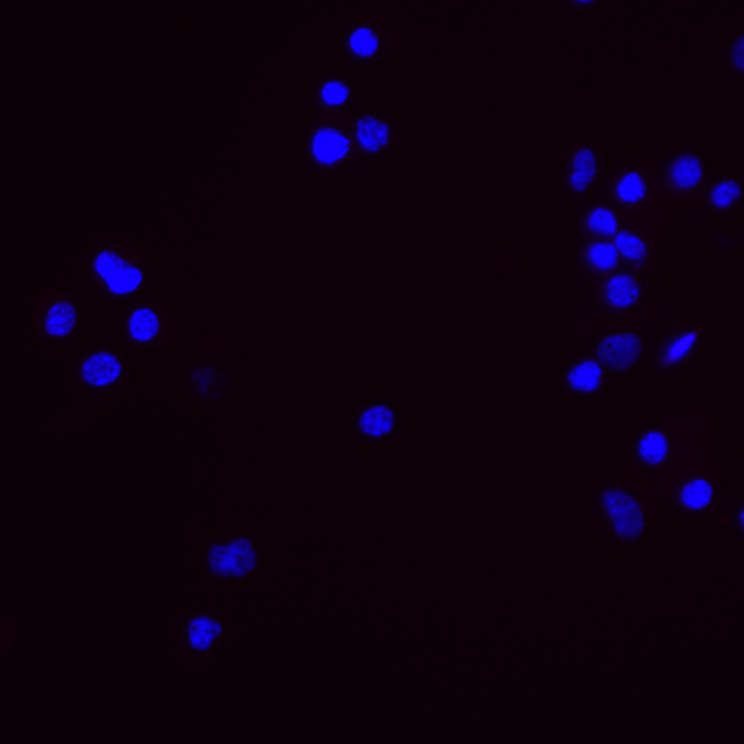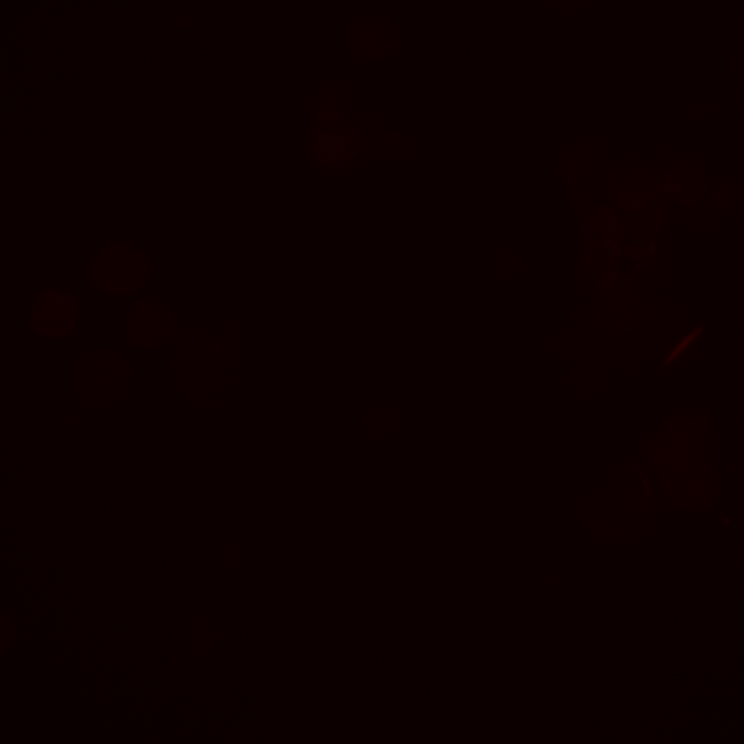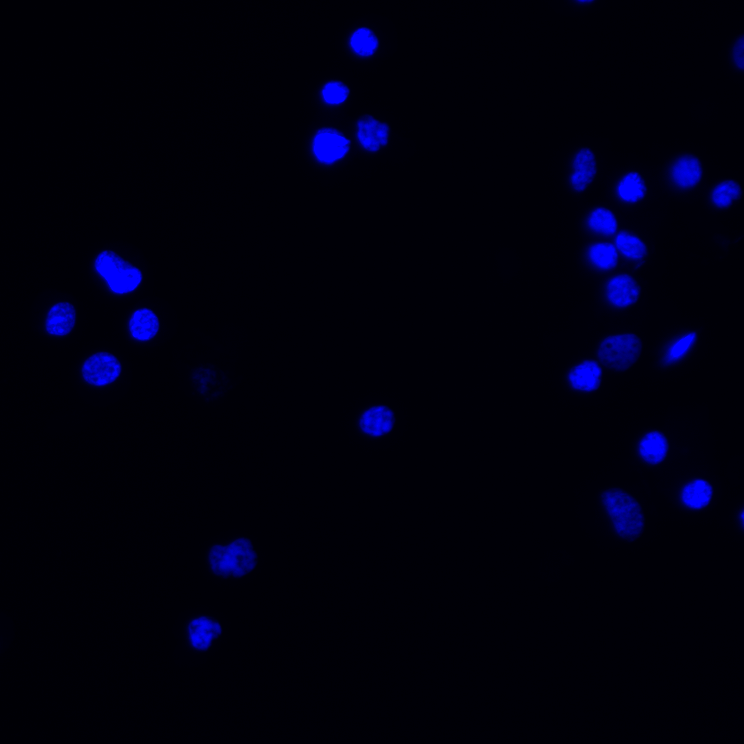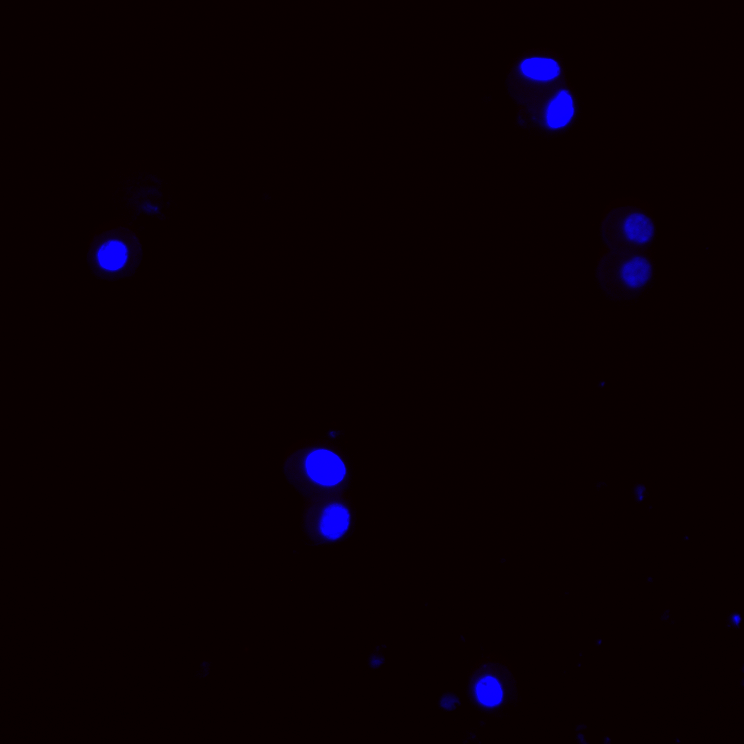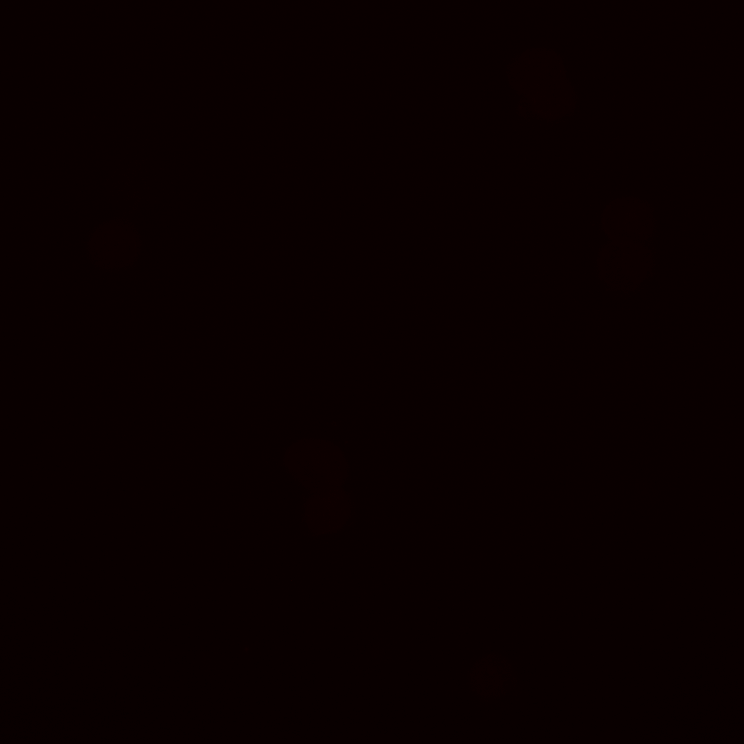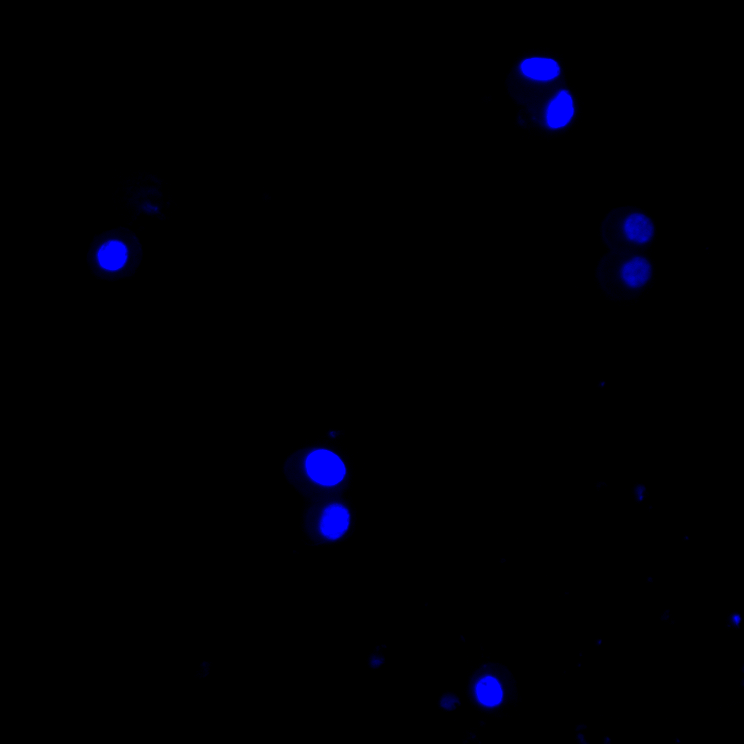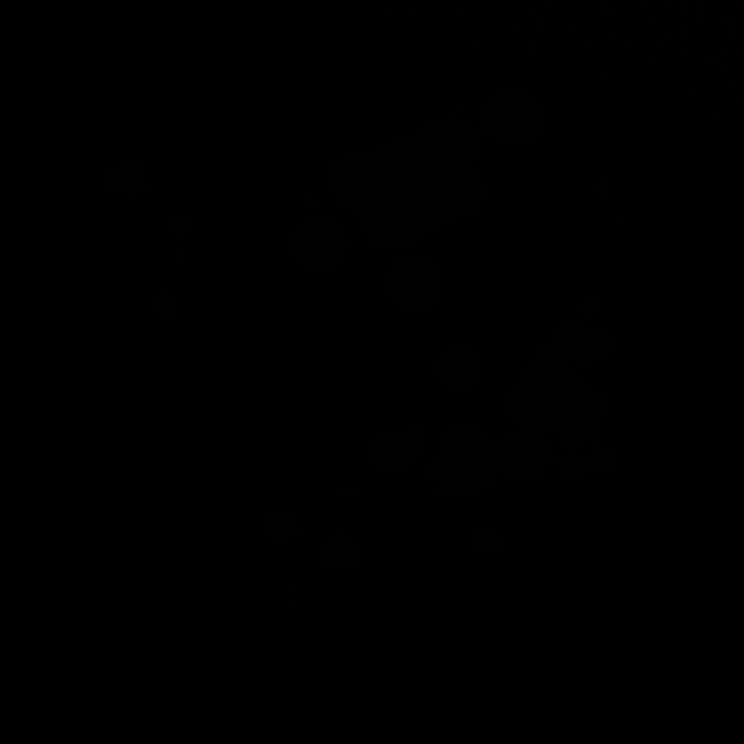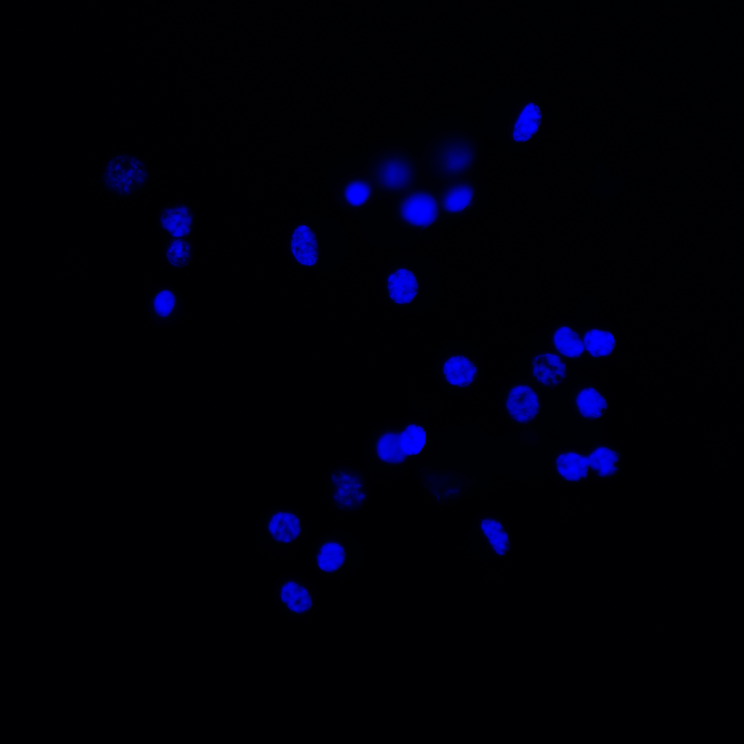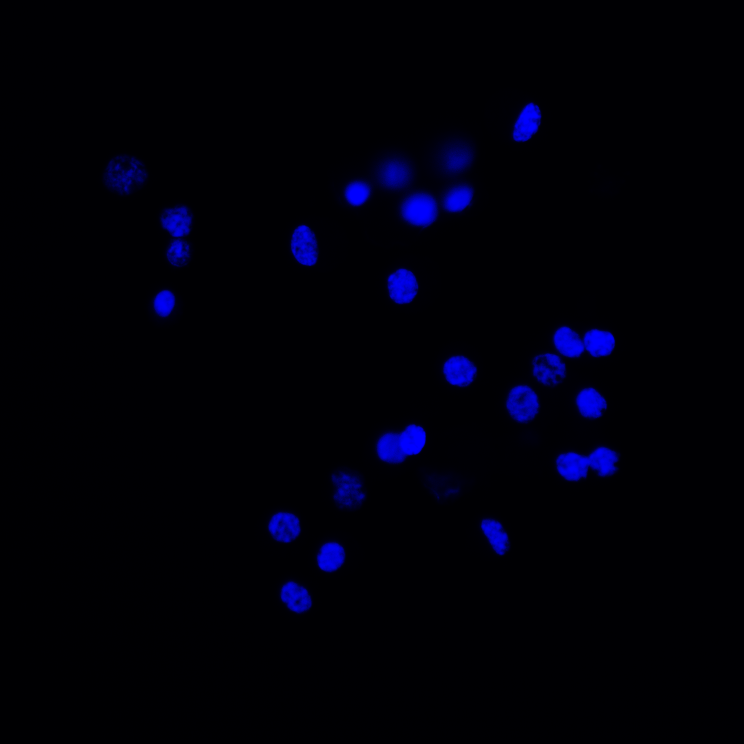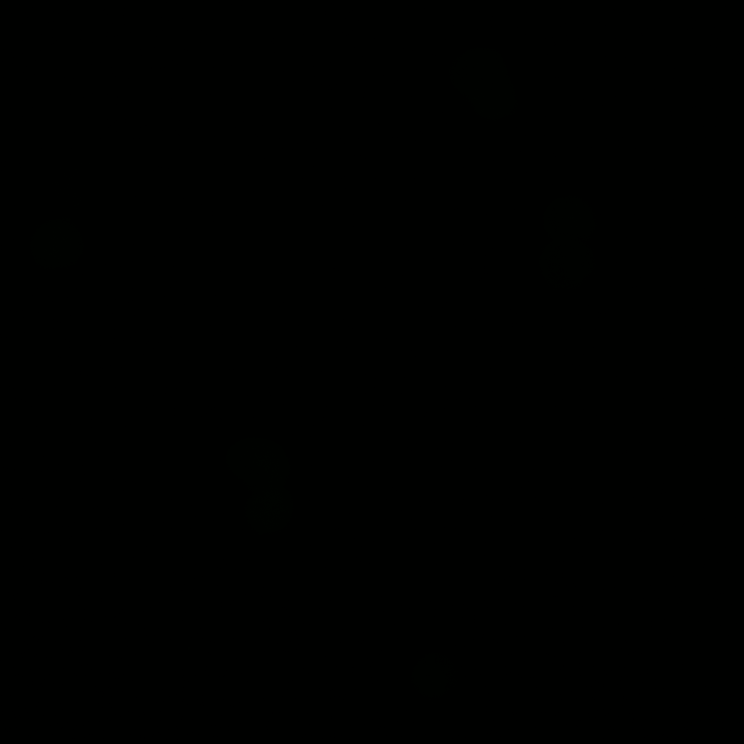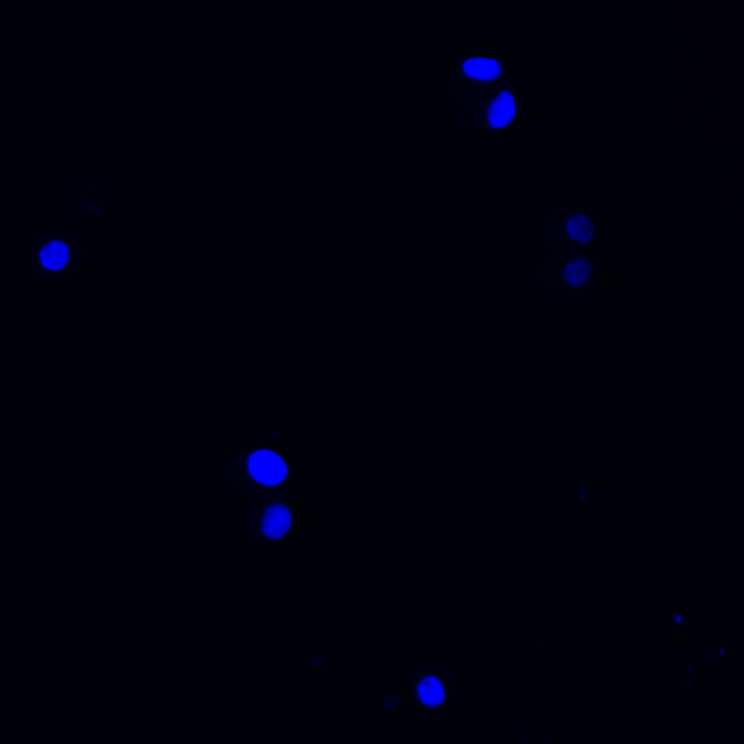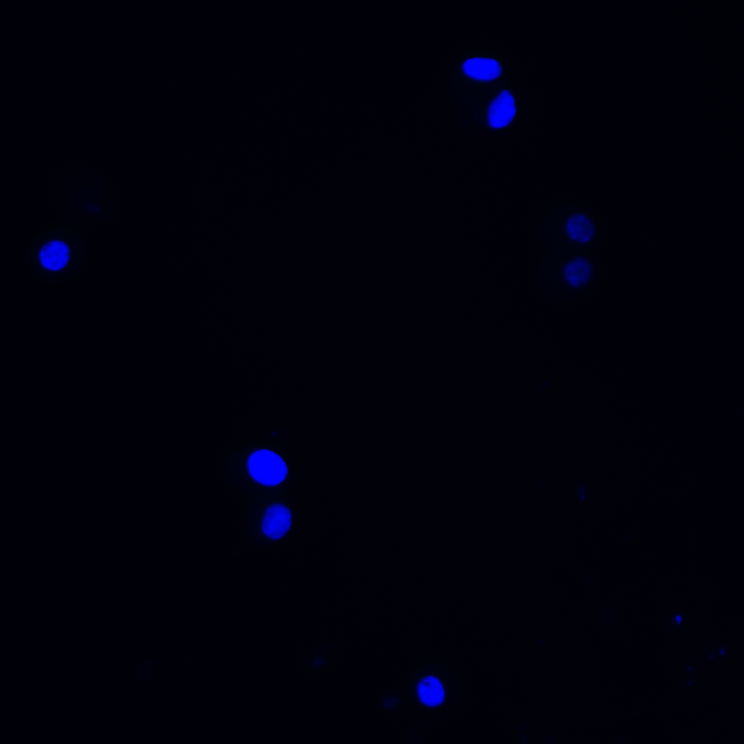 **DAPI**  **Merge**  **DAPI**  **Merge**  **A**  **B**  **C** |
| --- |

**Fig. S1. Control IgG staining.** ATII cell cytospins were obtained from control organ donors and stained with **(A)** anti-mouse (green), **(B)** anti-rabbit (red), and **(C)** anti-goat (red) IgG or secondary antibody by immunofluorescence (DAPI - blue, scale bar - 5µm, N = 3 lungs per group).

| **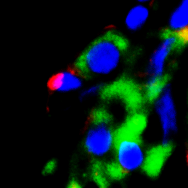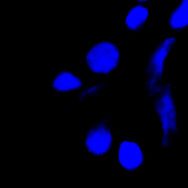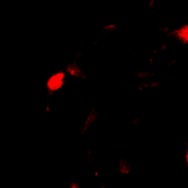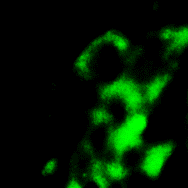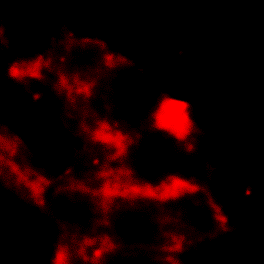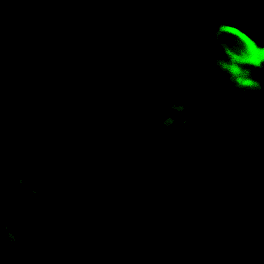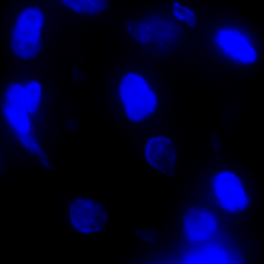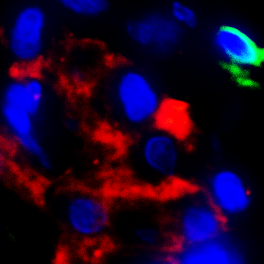**  **Merge**  **α-SMA**  **SP-C**  **DAPI**  **Control**  **IPF** |
| --- |

**Fig. S2. High α-SMA expression in IPF.** Lung tissue sections were obtained from control organ donors and IPF patients. They were stained with α-SMA (red), SP-C (green), and DAPI (blue) by immunofluorescence. The percentage of α-SMA+ cells is shown (scale bar - 5µm). Data are expressed as means ± SD; N = 3 lungs per group; *p*<0.001.

| 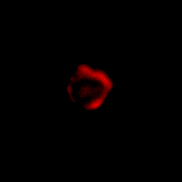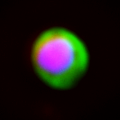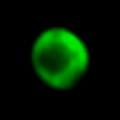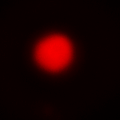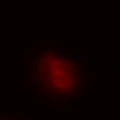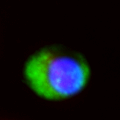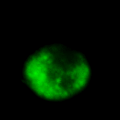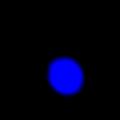 **H3K27me3**  **SP-C**  **DAPI**  **Merge**  **2kPa** 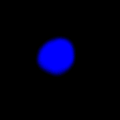 **50kPa**  **A** 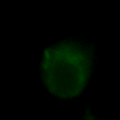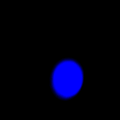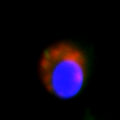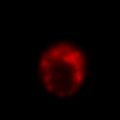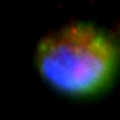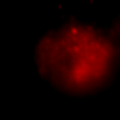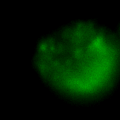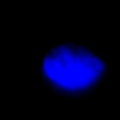 **H3K9me3**  **SP-C**  **DAPI**  **Merge**  **2kPa**  **50kPa**  **B** 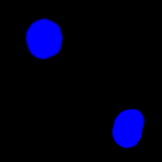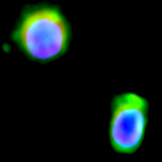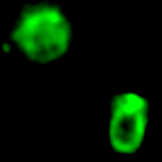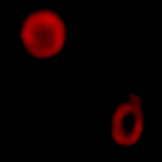 **Emerin**  **SP-C**  **DAPI**  **Merge**  **2kPa**  **50kPa**  **C** 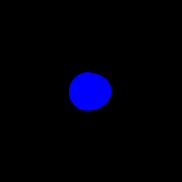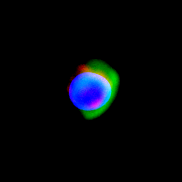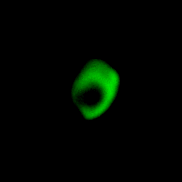 |
| --- |

**Fig. S3. Impact of mechanical stiffness on ATII cells.** ATII cells were obtained from control organ donors and cultured for 24h. **(A)** ATII cells were stained with H3K27me3 (red), SP-C (green), and DAPI (blue) by immunofluorescence. **(B)** ATII cells were stained for H3K9me3 (green), SP-C (red), and DAPI (blue) by immunofluorescence. H3K27me3 and H3K9me3 fluorescence intensity is shown. **(C)** Cultured ATII cells were stained with emerin (red), SP-C (green), and DAPI (blue) by immunofluorescence. Emerin fluorescence intensity and the number of cells with emerin discontinuity was quantified (scale bar - 10µm); N = 3 lungs per group; *p*<0.05.

**Fig. S4**. **mRNA expression of DNA damage-related genes in mechanical stiffness on ATII cells.** Control ATII cells were cultured on 2kPa and 50kPa PDMS gels for 48h. The mRNA expression of *PARP1,* *DNA Ligase IV, DJ-1, XRCC4, RAD50, MRE11,* *DNA Ligase III, SRX, TDP1, XLF,* and *NBS1* in ATII cells was detected by RT-PCR. Data are normalized to control and expressed as means ± SD; N = 3 lungs per group, *p*<0.05.
