# Supplementary material for: Stiffness-dependent alveolar type II cell senescence in idiopathic pulmonary fibrosis
Source: Cell Commun Signal. 2026 Apr 25;24:345. doi: 10.1186/s12964-026-02881-5 (PMC13248465; doi:10.1186/s12964-026-02881-5)
Supplement: Supplementary file 2 — Supplementary Material 2: Table. S1. Sequences of primers used for RT-PCR. [file 12964_2026_2881_MOESM2_ESM.docx]

**SUPPLEMENTARY MATERIAL**

**Stiffness-dependent alveolar type II cell senescence in idiopathic pulmonary fibrosis**

Chih-Ru Lin^1,2^, Khanutsanan Woranam^2,3^, Hassan Hayek^2,3^, Jonathan Jeger^2^, Loukmane Karim^2^, Beata Kosmider^2,3^, Rafal Kaminski^3,4^, Christopher W. Schultz^5^, Sudhir Bolla^6^, Nathaniel Marchetti^6^, Gerard J. Criner^6^, Karim Bahmed^2,3*^

^1^Department of Biochemistry, School of Medicine, College of Medicine, Kaohsiung Medical University, Kaohsiung, Taiwan

^2^Center for Inflammation and Lung Research, Lewis Katz School of Medicine, Temple University, Philadelphia, PA 19140, USA

^3^Department of Microbiology, Immunology, and Inflammation, Lewis Katz School of Medicine, Temple University, Philadelphia, PA 19140, USA

^4^Center for Neurovirology and Gene Editing, Lewis Katz School of Medicine, Temple University, Philadelphia, PA 19140, USA

^5^Department of Cancer and Cellular Biology, Lewis Katz School of Medicine, Temple University, Philadelphia, PA 19140, USA

^6^Department of Thoracic Medicine and Surgery, Lewis Katz School of Medicine, Temple University, Philadelphia, PA 19140, USA

*Corresponding author: Karim Bahmed, Ph.D.

Department of Microbiology, Immunology, and Inflammation

Center for Inflammation and Lung Research

Temple University

3500 N. Broad Street, Philadelphia, PA 19140

E-mail: [karim.bahmed@temple.edu](mailto:karim.bahmed@temple.edu)

**Table S1.** Sequences of primers used for RT-PCR.

| *Chr-10q* | F | 5’-GAATCCTGCGCACCGAGAT-3' |
| --- | --- | --- |
|  | R | 5'-CTGCACTTGAACCCTGCAATAC-3' |
| *Chr-15q* | F | 5’-CAGCGAGATTCTCCCAAGCTAAG-3’ |
|  | R | 5'- AACCCTAACCACATGAGCAACG -3' |
| *Chr-XP-YP* | F | 5’-GCAAAGAGTGAAAGAACGAAGCTT-3’ |
|  | R | 5'-CCCTCTGAAAGTGGACCAATCA-3' |
| *Chr-Xq-Yq* | F | 5’-GGAAAGCAAAAGCCCCTCTGAATG -3’ |
|  | R | 5'-ACCCTCACCCTCACCCTAAGC-3' |
| *DJ-1* | F | 5'-GTA GCC GTG ATG TGG TCA TTT-3' |
|  | R | 5’-CTG TGC GCC CAG ATT ACC T-3' |
| *DNA ligase III* | F | 5'-TCACTGGCGTGATGTAAGACA-3' |
|  | R | 5'-CCTGGAATGATAGAACAGGCTTT-3' |
| *DNA ligase IV* | F | 5’-AGCAAAAGTGGCTTATACGGATG-3' |
|  | R | 5'-TGAGTCCTACAGAAGGATCATGC-3' |
| *GAPDH* | F | 5’-GGAGCGAGATCCCTCCAAAAT-3' |
|  | R | 5’-GGCTGTTGTCATACTTCTCATGG-3’ |
| *Ku80* | F | 5'-GCACTGACAATCCCCTTTCTG -3' |
|  | R | 5’-TCAATGTCCTCCAGCAAATCAAA -3’ |
| *MRE11* | F | 5’-ATGCAGTCAGAGGAAATGATACG-3’ |
|  | R | 5'- CAGGCCGATCACCCATACAAT -3' |
| *NBS1* | F | 5'-CACTCACCTTGTCATGGTATCAG -3' |
|  | R | 5'-CTGCTTCTTGGACTCAACTGC -3' |
| *PARP1* | F | 5’- GCAGAGTATGCCAAGTCCAACAG-3’ |
|  | R | 5’-ATCCACCTCATCGCCTTTTC -3' |
| *RAD50* | F | 5'-TTTGGTTGGACCCAATGGGG -3' |
|  | R | 5'-CAGGAGGGAAATCTCCAGTACAA -3' |
| *SRX* | F | 5’-CAGGGAGGTGACTACTTCTACTC-3' |
|  | R | 5’-CAGGTACACCCTTAGGTCTGA-3' |
| *TDP1* | F | 5'-GCGAGAGGCTAAGGCTCAC-3' |
|  | R | 5'-TGTGTTCCAAACGCAATATCCAA-3' |
| *XLF* | F | 5'-CACTGATTCTACGGGTGCGAA -3' |
|  | R | 5’-CCTCACTTGGCACTGTAATGC -3’ |
| *XRCC4* | F | 5'-ATGTTGGTGAACTGAGAAAAGCA -3' |
|  | R | 5'-GCAATGGTGTCCAAGCAATAAC -3' |
